# Generation of late Mesozoic felsic volcanic rocks in the Hailar Basin, northeastern China in response to overprinting of multiple tectonic regimes

**DOI:** 10.1038/s41598-019-52181-x

**Published:** 2019-11-01

**Authors:** Zheng Ji, Qi-An Meng, Chuan-Biao Wan, De-Feng Zhu, Wen-Chun Ge, Yan-Long Zhang, Hao Yang, Yu Dong, Yan Jing

**Affiliations:** 1 College of Earth Sciences, Jilin University, Changchun, 130061 China; 2 Exploration and Development Research Institute, Daqing Oilfield Limited Company, Daqing, 163712 China

**Keywords:** Geochemistry, Geodynamics, Petrology, Tectonics, Volcanology

## Abstract

We performed zircon U–Pb age dating and geochemical analyses of late Mesozoic felsic volcanic rocks in the Hailar Basin, NE China, with the aim of eclucidating their emplacement ages, origin and geodynamic significance. The volcanic rocks consist of dacites, rhyolites and rhyolitic tuffs. Laser ablation–inductively coupled plasma–mass spectrometry zircon U–Pb dating results suggest that the rocks were erupted during the Late Jurassic–Early Cretaceous (161–117 Ma). They belong to the high-K calc-alkaline series and can be divided into two groups. Group I rocks are metaluminous to weakly peraluminous, contain low concentrations of heavy rare earth elements (HREEs) and high field strength elements (HFSEs), and have low zircon saturation temperatures (average 786 °C), all of which indicate an I-type affinity. In contrast, Group II rocks have higher HREE and HFSE concentrations and zircon saturation temperatures (average 918 °C), suggesting an A-type affinity. All the felsic volcanic rocks have positive ε_Hf_(t) values of 1.43–12.32 with two-stage model ages of 1110–401 Ma. Our data indicate that the I-type felsic volcanic rocks formed from magmas generated by partial melting of a dominantly juvenile mica-bearing K-rich basaltic lower crust, whereas the A-type felsic volcanic rocks originated from the partial melting of a dry mafic–intermediate middle–lower crust that was dehydrated but not melt depleted. Based on the present results and previous research, we propose that the Late Jurassic I- and A-type felsic volcanic rocks in the Hailar Basin were formed in a post-collisional environment related to break-off of the subducted oceanic slab of the Mongol–Okhotsk Ocean and the subsequent gravitational collapse of the orogenically-thickened crust after closure of the ocean. In contrast, the Early Cretaceous I- and A-type felsic volcanic rocks were erupted in an extensional setting related to rollback of the subducted Paleo-Pacific Plate.

## Introduction

**Figure 1:**
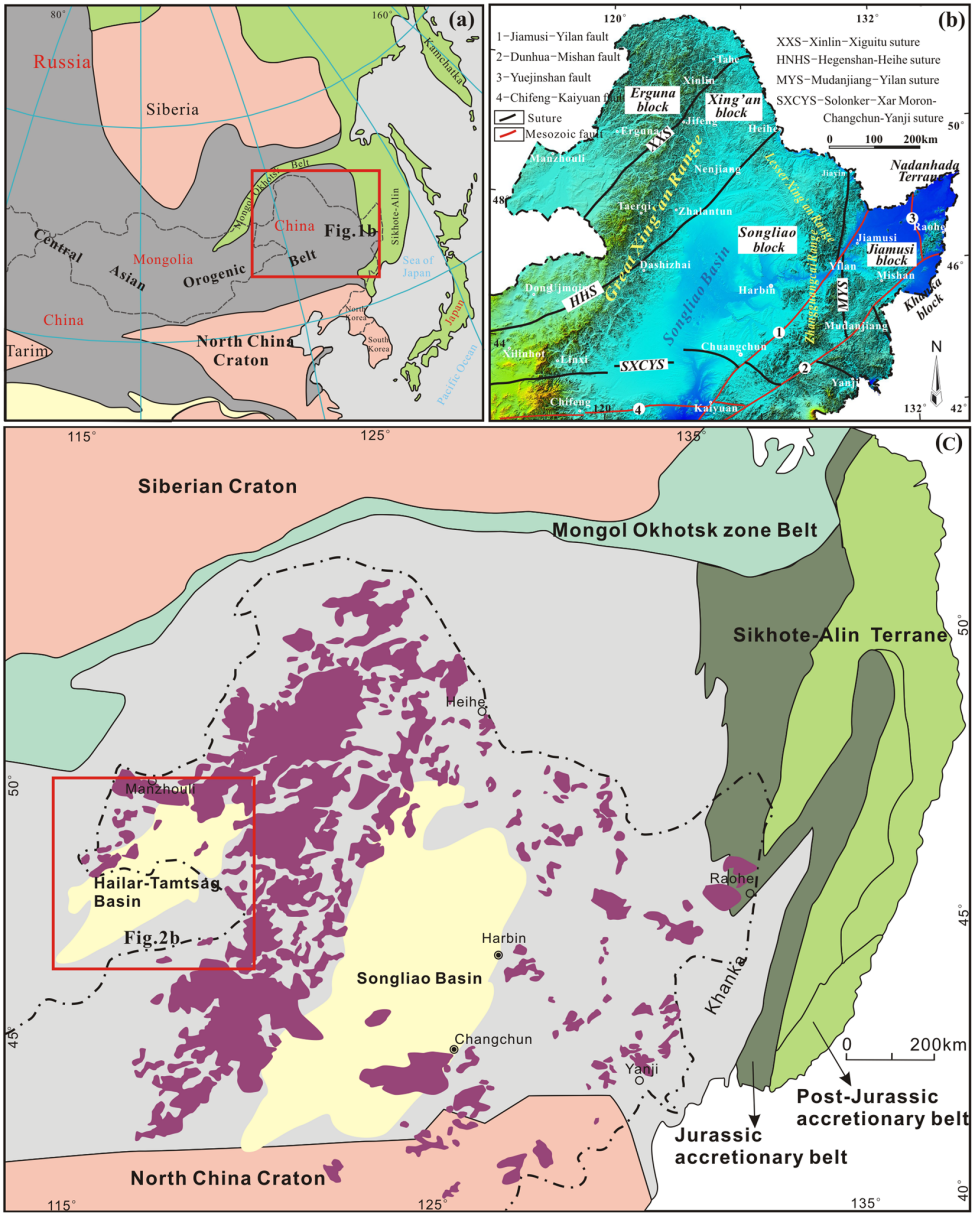
(**a**) General map of the Central Asian Orogenic Belt, showing the location of northeastern China (modified after Safonova^12^). (**b**) Tectonic division of northeastern China (modified after Liu^3^). (**c**) Map showing the distribution of Mesozoic volcanic rocks in NE China (modified after Xu^8^).

Northeast China contains two contrasting Phanerozoic orogenic systems: the easternmost part of the Central Asian Orogenic Belt (CAOB) and the western part of the circum-Pacific Orogenic Belt^1–4^ (Fig. 1a). The region is considered to have evolved through the accretion of variably-sized continental fragments and juvenile oceanic material (e.g., ophiolites, island arcs, oceanic islands, seamounts, and oceanic plateaux) throughout the Phanerozoic, forming a complex orogenic collage^5,6^ (Fig. 1b). The initial tectonic evolution of NE China was controlled by closure of the Paleo-Asian Ocean, which led to the collision of the North China and Siberian cratons at the end of the Permian and the beginning of the Early Triassic, thus building the renowned CAOB^7^. Younger tectonomagmatic events were related to closure of the Mongol–Okhotsk Ocean (MOO) and subduction of the Pacific Plate^8–10^. These events resulted in large-scale late Mesozoic magmatism and the development of accretionary complexes along the eastern margin of the Asian continental^11–14^ (Fig. 1c).

The complex interactions of multiple plates and the superimposition of tectonic regimes mean that the origin and geodynamic significance of the late Mesozoic magmatism in NE China remain controversial^7,8,15^. Meng^16^ invoked break-off of the Mongol–Okhotsk oceanic slab in conjunction with gravitational collapse after closure of the ocean to explain the widespread occurrence of late Mesozoic extensional basins, volcanic rocks, and metamorphic core complexes in NE China. Nevertheless, Zhang^11,17^ attributed the inland migration of Jurassic magmatism to low-angle subduction of the Pacific Plate, and the trenchwards-younging trend of the Early Cretaceous magmatism to subduction-induced migrating delamination or slab rollback. Furthermore, some researchers have recently emphasised the mutual impacts of the closure of the MOO and the subduction of the Pacific Plate, but the respective spatial–temporal effects of these two tectonic influences have not been fully ascertained. Recently, Li^15^ explained the intense late Mesozoic tectonomagmatic events in NE China in terms of a water-fluxed melting model, in which the trapped fossil Paleo-Asian oceanic slab in the mantle transition zone provided water for melting of the crust and lithospheric mantle.

Hundreds of late Mesozoic extensional basins developed in NE China, covering an area in excess of 3,000,000 km2, and they were filled with large-scale late Mesozoic volcano-sedimentary successions^8,16,18,19^. Although the late Mesozoic volcanic rocks have been well-studied in outcrop, there have been few studies of the subsurface volcanic succession in these basin fills. The Hailar Basin (HB), an important part of the late Mesozoic extensional basin system in NE China, is characterized by episodic rifting, dramatic magmatism, and thick terrestrial sediments, which provides crucial opportunities to understand late Mesozoic magma-tectonic evolution of NE China. However, there is a lack of isotope chronological and geochemical data for the subsurface volcanic rocks, which restricts our understanding of the origin of this dramatic and widespread late Mesozoic magmatic activity in NE China. In this paper, we present new whole-rock geochemical, geochronological and zircon Lu–Hf isotopic data for the late Mesozoic felsic volcanic rocks in the HB. These new data have helped us to unravel the origin of these rocks and develop a tectonomagmatic model for the generation of the voluminous late Mesozoic magmatic rocks of NE China.

## Geological Setting

NE China is located in the eastern segment of the CAOB that separates the North China Craton from the Siberia Craton (Fig. 1a), and comprises a collage of microcontinents of different types and derivation^3,7^. The western part of NE China is made up of the Erguna, Xing’an and Songliao blocks, which were amalgamated gradually as a result of the Paleozoic evolution and closure of the Paleo-Asian Ocean^6,7,20^. The eastern part of NE China contains the Jiamusi–Khanka and Nadanhada blocks. The former is considered to be an exotic fragment derived from Precambrian Gondwana^21^, whereas the Nadanhada Block as well as the Sikhote–Alin Terrane of the Russian Far East and the Tamba–Mino–Ashio Terrane of Japan represent accretionary prisms or basins that formed as a result of late Mesozoic subduction of the Paleo-Pacific Plate^4,12^. The Mesozoic tectonic framework in the northwest of NE China was overprinted by the closure of the MOO, which involved thrusting, folding, magmatism, and the formation of the Mongol–Okhotsk Suture Belt^9,16^. Large-scale eruptive and intrusive magmatic activity, predominantly intermediate to felsic with lesser mafic composition, occurred during the late Mesozoic in response to closure of the MOO and subduction of the Paleo-Pacific Plate^9,22^. The products of this magmatic activity occur extensively in various fault-controlled basins and ranges, such as the HB and Great Xing’an Range (GXR) in the west, and the Songliao Basin (SB) and Lesser Xing’an–Zhangguangcai ranges (LXZR) in the east^7,8^ (Fig. 1c).

**Figure 2:**
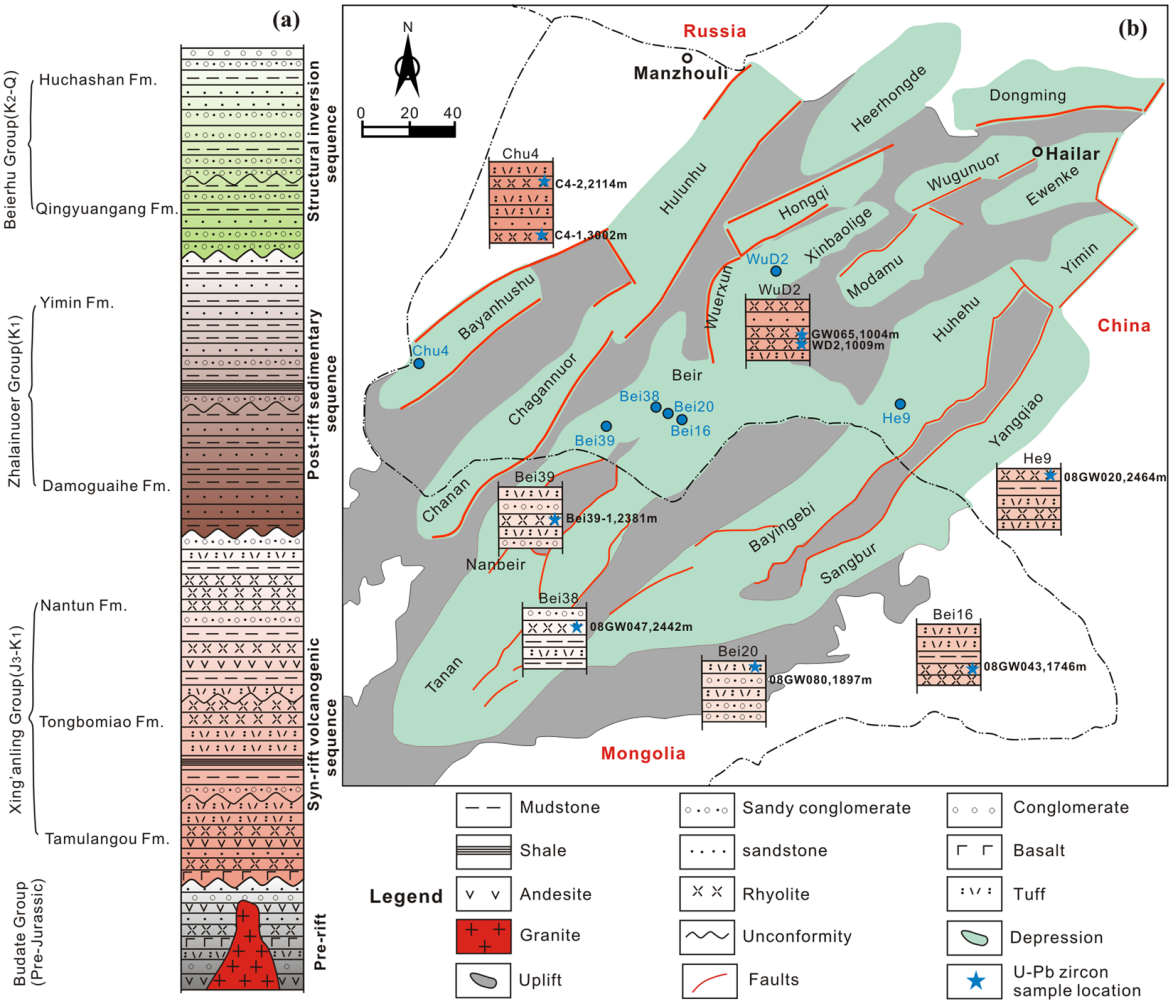
(**a**) Conceptual stratigraphic column for the Hailar Basin (modified after Wan^23^). (**b**) Geological map showing the distribution of boreholes in the Hailar Basin and the locations of samples analysed during this study.

The HB is a Mesozoic–Cenozoic petroliferous fault-bounded volcanic basin in the westernmost region of NE China, and it extends into northeastern Mongolia as the Tamtsag Basin^23–25^. Its western part belongs to the Erguna Block, while the eastern part belongs to the Xing’an Block. The NE–SW-trending HB exhibits two zones of uplift and three areas of depression that are subdivided into 16 sags^23^ (Fig. 2b). The stratigraphic succession of the HB comprises the pre-Jurassic Budate Group, the Upper Jurassic–Lower Cretaceous Xing’anling and Zhalainuoer groups, and the Upper Cretaceous–Paleocene Beierhu Group^24^ (Fig. 2a). The Budate Group, which consists of conglomerate and sandstone with interlayered volcanic rocks, is considered to represent the basement of the basin^26^. Meng^26^ reported zircon U–Pb ages of 295–356 Ma for volcanic rocks from the Budate Group, and they suggested these volcanic rocks formed during the late Paleozoic rather than the early Mesozoic, as had previously been thought. The Xing’anling Group contains a syn-rift succession that is subdivided from bottom to top into the Tamulangou, Tongbomiao and Nantun formations^24^. The Tamulangou Formation consists of lavas with interbedded volcaniclastic rocks and coal beds. It is discontinuously distributed in the HB and was deposited during an initial rifting stage^25^. The dominant Tongbomiao and Nantun formations are composed of lacustrine and alluvial mudstone, siltstone, sandstone, and conglomerate. Moreover, the Tongbomiao and Nantun formations contain abundant felsic volcanic rocks that were produced during the climax of volcanism and rifting^23^. The overlying Zhalainuoer Group is a post-rift succession that was deposited under fluvial–deltaic and lacustrine conditions.

The felsic volcanic samples were collected from the Tamulangou, Tongbomiao and Nantun formations in the HB. Details of the locations (including well locations and depth) are provided in Table S1 and Fig. 2b. The felsic volcanic rocks are mainly rhyolites, dacites, and rhyolitic tuffs (Fig. S1). The rhyolites are either massive or display flow structures, and they are porphyritic with phenocrysts of plagioclase, alkali feldspar and quartz in an aphanitic or glassy groundmass. The dacites are massive in structure and porphyritic with phenocrysts of quartz, plagioclase and biotite in a groundmass of aphanitic felsic minerals and glass. The rhyolitic tuffs are massive in structure, and they consist of volcanic ash and crystal fragments of quartz, feldspar and biotite.

## Results

### Zircon U–Pb and Lu–Hf isotope

Nine representative samples were selected for zircon U–Pb LA–ICP–MS dating (Table S2). Some of the U–Pb dating spots in the zircons from samples C4-1, C4-2, WD2, 08GW020, 08GW043, 08GW047 and Bei39-1 were also chosen for zircon Hf isotope analyses (Table S3). The zircon grains collected for dating were mostly colourless, transparent, euhedral–subhedral crystals that exhibit oscillatory growth zoning in cathodoluminescence (CL) images (Fig. S2). They have high Th/U ratios of 0.1–2.2, point to a magmatic origin.

**Figure 3:**
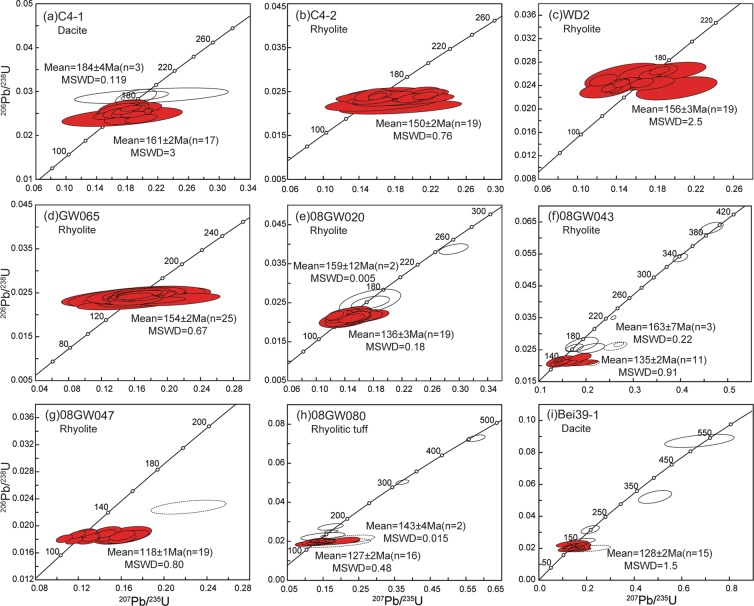
Zircon U–Pb concordia diagrams showing LA–ICP–MS ages obtained during this study, including weighted mean ages and MSWD values.

**Figure 4:**
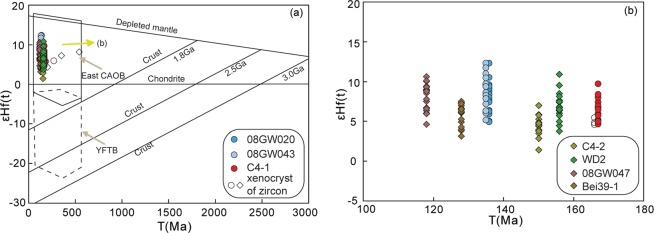
(**a**) Zircon ε_Hf_(t) vs. U–Pb age diagram for the late Mesozoic felsic volcanic rocks in the Hailar Basin. CAOB = Central Asian Orogenic Belt; YFTB = Yanshan Fold-and-Thrust Belt. The distributions of the east CAOB and YFTB are from Yang^84^. (**b**) Distribution of seven samples on the ε_Hf_(t) vs. U–Pb age diagram, enlarged from the area indicated by the square in (**a**).

Dacite sample C4-1 was collected from borehole Chu4 in the Bayanhushu Depression of the HB. Twenty concordant analyses have ^206^Pb/^238^U ages of 185 to 152 Ma, with two populations yielding mean ages of 161 ± 2 Ma (MSWD = 3; n = 17) and 184 ± 4 Ma (MSWD = 0.119; n = 3) (Fig. 3a). The first population (161 ± 2 Ma) represents the crystallisation age of the dacite, whereas the second possibly represents the crystallisation ages of inherited or captured zircons entrained by the dacite magma. Seventeen zircon crystals from the sample have ε_Hf_(t) values of +4.7 to +9.7 and T_DM2_ ages of 916–592 Ma (Fig. 4). One captured zircon crystal (184 Ma) yielded a ε_Hf_(t) value of +5.7 and a T_DM2_ age of 848 Ma.

Rhyolite sample C4-2 was collected from borehole Chu4 in the Bayanhushu Depression of the HB. Nineteen analyses produced concordant to near-concordant ^206^Pb/^238^U ages ranging from 156 to 136 Ma, defining a weighted mean ^206^Pb/^238^U age of 150 ± 2 Ma (MSWD = 0.76, n = 19; Fig. 3b), which is interpreted to represent the crystallisation age of the rhyolite. Nineteen zircon crystals from the rhyolite have ε_Hf_(t) values of +1.4 to +7.0 and T_DM2_ ages of 1110–752 Ma (Fig. 4).

Two samples of rhyolite (WD2 and GW065) were collected from borehole WuD2 in the Wuerxun Depression of the HB. Nineteen analyses from sample WD2 produced concordant to near-concordant ^206^Pb/^238^U ages ranging from 169 to 149 Ma, defining a weighted mean ^206^Pb/^238^U age of 156 ± 3 Ma (MSWD = 2.5, n = 19; Fig. 3c). Analyses of 19 zircon grains from rhyolite WD2 yielded ε_Hf_(t) values of +3.8 to +10.9 and T_DM2_ ages of 963–508 Ma. Dating results for 25 zircon grains from sample GW065 gave concordant and consistent ages, with a weighted mean ^206^Pb/^238^U age of 154 ± 2 Ma (MSWD = 0.67, n = 25; Fig. 4).

Rhyolite sample 08GW020 was collected from borehole He9 in the Huhehu Depression of the HB. Twenty-one analyses produced ^206^Pb/^238^U ages ranging from 160 to 130 Ma and two population that yield mean ages of 136 ± 3 Ma (MSWD = 0.18; n = 19) and 159 ± 12 Ma (MSWD = 0.005; n = 2) (Fig. 3e). The first population (136 ± 3 Ma) represents the crystallisation age of the rhyolite, whereas the second population, together with another ^206^Pb/^238^U age of 245 Ma, possibly represents the crystallisation ages of inherited or captured zircons entrained by the rhyolite magma. Nineteen zircon crystals from the rhyolite have ε_Hf_(t) values of +5.0 to +12.3 and T_DM2_ ages of 874–401 Ma (Fig. 4). One captured zircon with an age of 245 Ma yielded a ε_Hf_(t) value of +5.7 and a T_DM2_ age of 913 Ma.

Rhyolite sample 08GW043 was collected from borehole Bei16 in the Beier Depression of the HB. Three analyses was excluded from the calculations because of high discordancy. The ^206^Pb/^238^U ages yielded by 17 spot analyses range from 395 to 131 Ma, with two populations that yield mean ages of 135 ± 2 Ma (MSWD = 0.91; n = 11) and 163 ± 7 Ma (MSWD = 0.22; n = 3) (Fig. 3f) as well as three single zircon ages of 395, 337, and 221 Ma. The youngest age population of 135 Ma is considered to represent the timing of crystallisation of the rhyolite. Eighteen zircon Lu–Hf isotopic analyses were conducted on this sample, with 15 analyses yielding ε_Hf_(t) values of +5.2 to +12.3 and T_DM2_ ages of 860–403 Ma (Fig. 4). The other three analyses of captured zircons (166 Ma) gave ε_Hf_(t) values of +4.6 to +5.5 and T_DM2_ ages of 918–864 Ma.

Rhyolite sample 08GW047 was collected from borehole Bei38 in the Beier Depression of the HB. One analysis was excluded from the calculations because of high discordancy. The remaining 19 data points defined a weighted mean ^206^Pb/^238^U age of 118 ± 1 Ma (MSWD = 0.80, n = 19; Fig. 3g), which is interpreted as the time of crystallisation of the rhyolite. Eighteen zircon crystals from rhyolite 08GW047 gave ε_Hf_(t) values of +6.0 to +10.6 and T_DM2_ ages of 879–498 Ma (Fig. 4).

Rhyolitic tuff sample 08GW080 was collected from borehole Bei20 in the Beier Depression of the HB. Two analyses was excluded from the calculations because of high discordancy. Eighteen analytical spots defined a concordia age group of 127 ± 2 Ma (MSWD = 0.48, n = 16) (Fig. 3h), which is considered to represent the timing of eruption. The remaining six spots gave one age group at 143 ± 4 Ma (MSWD = 0.015, n = 2) and four older inherited or captured zircon ages of 453, 315, 172, and 158 Ma.

Rhyolite sample Bei39-1 was collected from borehole Bei39 in the Beier Depression of the HB. One analysis was excluded from the calculations because of high discordancy. Fifteen analytical spots defined a concordia age group of 128 ± 2 Ma (MSWD = 1.5, n = 15) (Fig. 3i), which is considered to represent the timing of crystallisation of the rhyolite. The remaining four spots gave four older inherited or captured zircon ages of 539, 326, 199, and 153 Ma. Sixteen zircon crystals from the sample gave ε_Hf_(t) values of +3.2 to +7.6 and T_DM2_ ages of 981–701 Ma (Fig. 4). Another three captured zircon crystals (539–199 Ma) yielded ε_Hf_(t) values of +4.4 to +8.1 and T_DM2_ ages of 981–894 Ma.

### Major and trace elements

**Figure 5:**
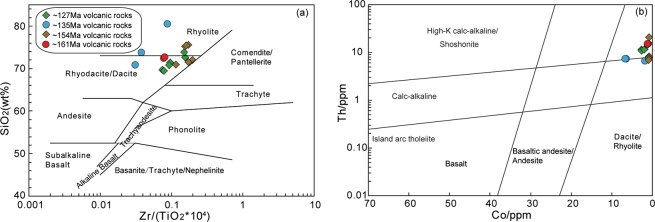
Plots of (**a**) SiO_2_ vs. Zr/TiO_2_ (after Winchester^85^) and (**b**) Th vs. Co (after Hastie^88^) for late Mesozoic felsic volcanic rocks in the Hailar Basin.

The whole-rock major and trace element compositions determined for the Late Jurassic–Early Cretaceous felsic volcanic rocks in the HB are provided in Table S4. The rocks display variations in composition from dacite to rhyolite, and they all plot consistently in the sub-alkaline field of the SiO_2_–Zr/TiO_2_ diagram (Fig. 5a). On the Th vs. Co diagram, all these rocks fall into the high-K calc-alkaline field (Fig. 5b). These volcanic rocks can be subdivided into two groups, according to their petrological and geochemical features, as follows.

#### Group I

**Figure 6:**
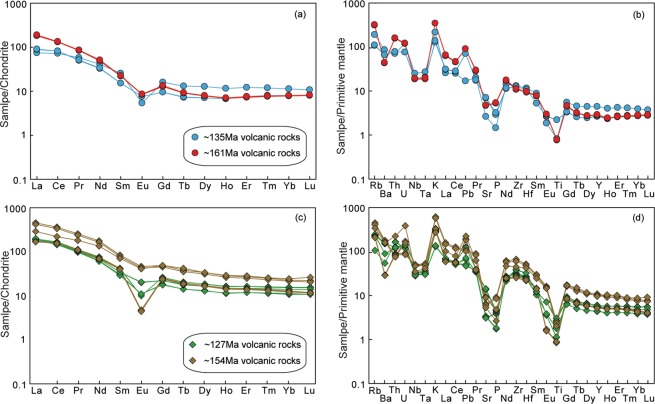
Chondrite-normalised REE patterns (a,c) and primitive-mantle-normalised trace element spidergrams (**b,d**) for late Mesozoic felsic volcanic rocks in the Hailar Basin. Normalizing values are from Sun^86^.

This group comprises the ~161 Ma and ~135 Ma felsic volcanic rocks, which have high SiO_2_ (70.73–80.35 wt.%) contents, TiO_2_ = 0.16–0.45 wt.%, Al_2_O_3_ = 8.38–12.49 wt.%, total Fe_2_O_3_ = 0.50–2.24 wt.%, MgO = 0.42–2.12 wt.%, CaO = 0.15–0.91 wt.%, P_2_O_5_ = 0.01–0.04 wt.%, Na_2_O = 0.41–3.14 wt.%, K_2_O = 2.68–5.63 wt.%, and K_2_O/Na_2_O = 1.27–8.39. With the exception of one sample (08GW020), the A/CNK (molar ratio of Al_2_O_3_/(CaO + Na_2_O + K_2_O)) values of these volcanic rocks fall within the range of 0.97–1.13, indicating their metaluminous to weakly peraluminous character. These rocks have total rare earth element (REE) contents of 101–173 ppm, and their chondrite-normalised REE patterns are characterised by a relative enrichment in light REEs ((La/Yb)_N_ = 6.19–22.25) and clearly negative Eu anomalies ((Eu/Eu*)_N_ = 0.26–0.62) (Fig. 6a). In the primitive mantle (PM)-normalised trace element diagram, these samples display positive Rb, Th and U anomalies and negative Nb, Ta, P and Ti anomalies (Fig. 6b).

#### Group II

This group comprises the ~154 Ma and ~127 Ma felsic volcanic rocks, which have high concentrations of SiO_2_ (69.43–75.53 wt.%) and K_2_O (3.10–8.22 wt.%), variable concentrations of Al_2_O_3_ (11.05–16.10 wt.%), Na_2_O (0.26–3.57 wt.%), and total Fe_2_O_3_ (0.22–1.30 wt.%), and low contents of MgO (0.17–0.80 wt.%), CaO (0.05–0.78 wt.%), and P_2_O_5_ (0.01–0.06 wt.%). This group of volcanic rocks is peraluminous with high A/CNK (>1) and A/NK (>1) values. They are characterised by high concentrations of incompatible trace elements such as Rb (68–289 ppm), Ga (18–32 ppm), Nb (20–36 ppm), La (41–110 ppm), Ce (90–225 ppm) and Y (20–50 ppm), and they have high Zr contents (286–740 ppm) and Zr/Nb ratios (8.51–21.55), reflecting their original peralkaline character (Leat *et al*., 1986). The samples show relatively steep REE patterns ((La/Yb)_N_ = 11.15–18.74), with high total REE abundances (192–492 ppm) and a wide range of negative Eu anomalies (0.14–0.71) (Fig. 6c). Positive anomalies of Rb, Th, Zr and Hf and negative anomalies of Ba, Nb, Ta, P and Ti are observed in the primitive mantle (PM)-normalised trace element diagram (Fig. 6d).

## Discussion

### Late Mesozoic volcanism in the HB and adjacent areas

**Figure 7:**
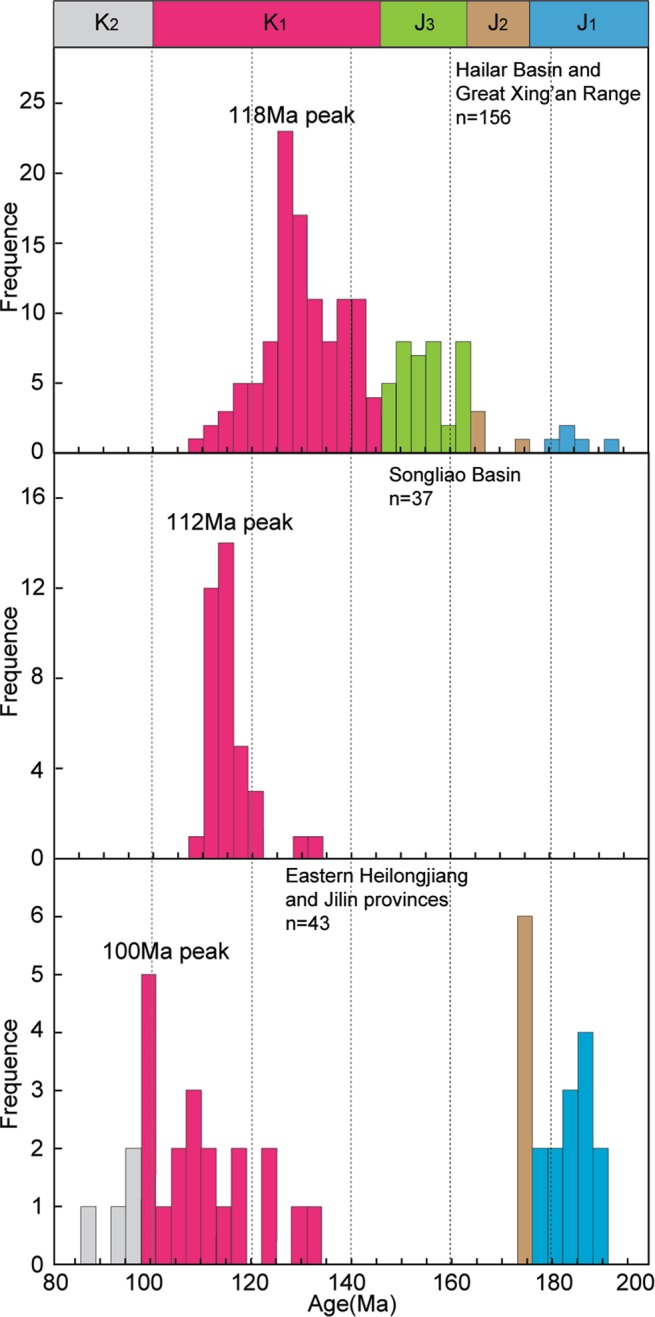
Probability plots of the U–Pb ages of zircons from Mesozoic volcanic rocks in NE China. Data sources are from Table S1.

The HB is filled with large volumes of late Mesozoic volcano-sedimentary material, which not only records the stratigraphic, sedimentary and structural evolution of the HB, but also provides constraints on the late Mesozoic magmatic–tectonic evolution of NE China. It has previously been suggested on the basis of geochronological studies that the late Mesozoic volcano-sedimentary successions in the HB spanned the period from the Late Jurassic to the Early Cretaceous, corresponding to the Xing’anling Group that comprises (from bottom to top) the Tamulangou, Tongbomiao and Nantun formations^23–25^. However, the age constraints are based mainly on rock associations and plant fossil assemblages, with few high-precision isotopic age data for the intercalated volcanic rocks within the stratigraphic units of the basin. For example, Li^27^ reported the Late Jurassic trachyandesites (~151 Ma) and the late Early Cretaceous rhyolitic tuffs (119–117 Ma) in the Hongqi Depression, and the Late Jurassic rhyolites (149–145 Ma) in the Hulunhu Depression; Zhao^28^ reported the Early Cretaceous volcanic rocks (128–120 Ma) in the Bayanhushu Depression. High-resolution isotopic age data of volcanic interlayers help to constrain the absolute ages of the strata, but the existing age data are inadequate to support stratigraphic classifications or determine the timing of volcanism in the HB and adjacent areas. Therefore, in this study we obtained LA–ICP–MS zircon U–Pb ages for nine samples of the late Mesozoic volcanic rocks in the HB. Zircon crystals from the samples have typical oscillatory growth zoning, indicative of a magmatic origin. Therefore, the obtained zircon U–Pb ages probably represent the timing of the formation of these volcanic rocks. Our new isotopic age data, combined with previous geochronological data^27,28^, indicate that the Mesozoic volcanic rocks in the HB formed mainly during the Late Jurassic to Early Cretaceous, with ages of 161 to 117 Ma. A compilation of available age data for volcanic rocks in the HB and the GXR shows that volcanism in the region was continuous from the Late Jurassic to the Early Cretaceous. This is in contrast to the striking lull in magmatic activity from the Late Jurassic to the early Early Cretaceous in the SB, LXZR, and eastern Heilongjiang–Jilin provinces (EHJP)^8^ (Fig. 7). Furthermore, the Early Cretaceous climax of volcanic activity displays a southeastwards younging trend in NE China, similar to the trend reported for metamorphic core complexes^29^.

### Petrogenesis

#### Genetic types and fractionation processes

**Figure 8:**
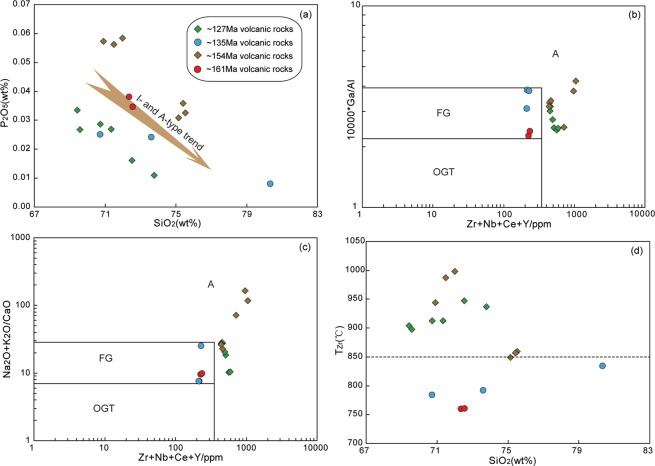
Plots of (**b**) P_2_O_5_ versus SiO_2_, (**b**) 10,000 × Ga/Al versus Zr + Nb + Ce + Y (after Whalen^30^), (**c**) (Na_2_O + K_2_O)/CaO versus Zr + Nb + Ce + Y (after Eby^87^), and (**d**) T_Zr_ versus SiO_2_ for late Mesozoic felsic volcanic rocks in the Hailar Basin. FG = highly fractionated I-type; OGT = unfractionated I-, S- and M-types; A = A-type.

Granites and felsic volcanic rocks are commonly divided into I-, S-, M- and A-types, based mainly on the nature of their protolith or tectonic affinities^30,31^. The studied felsic volcanic rocks in the HB have high SiO_2_ (69.43–80.35 wt.%) contents, low Cr (0.52–27.68 ppm), Co (0.10–6.65 ppm), Ni (0.08–15.37 ppm) and MgO (0.17–2.12 wt.%) contents, and contain inherited zircons. These observations suggesting that they do not belong to an M-type suite derived from a mantle source. Although Whalen^30^ defined an A/CNK value of 1.1 as the boundary between S-type granites and other types (i.e., I- and A-type granites), it is difficult to distinguish S-type from highly fractionated I- and A-type granites because of their overlapping geochemical features^30^. Our volcanic rock specimens are characterised by high Rb, Th and U contents, high Rb/Sr ratios, low TiO_2_, CaO and P_2_O_5_ contents, and low K/Rb ratios, indicating that the magmas were highly fractionated. Consequently, it is not possible to determine whether these volcanic rocks belong to S-type or other types based solely on A/CNK values. Based on the solubility of apatite in different granitic melts, and therefore the contrasting variation trends of P_2_O_5_ in S-, I- and A-type granites, Chappell^31^ and Li^32^ suggested that P_2_O_5_ contents and the correlation between P_2_O_5_ and SiO_2_ are important in determining the magmatic affinity of a granite, because apatite reaches saturation in metaluminous–weakly peraluminous and peralkaline granitic melts but is highly soluble in strongly peraluminous melts^33^. A negative correlation between P_2_O_5_ and SiO_2_ in our volcanic rock specimens strongly suggests that the volcanic rocks are not S-type but are A- or I-types (Fig. 8a), as supported by the lack of primary Al-rich minerals such as muscovite or garnet.

In general, A-type granites are characterised by enrichments in high field strength elements (HFSEs) and REE contents, high alkali (Na_2_O + K_2_O) concentrations and Ga/Al ratios, but low concentrations of MgO and CaO^30,34–36^. The *Group I* volcanic rocks have significantly higher REEs and HFSEs contents than those of the *Group II* volcanic rocks. Furthermore, in the 10,000 × Ga/Al and (Na_2_O + K_2_O)/CaO versus Zr + Nb + Ce + Y diagrams (Fig. 8b,c), the volcanic samples of *Group I* plot in the highly fractionated I-type field, whereas those of *Group II* fall in the A-type field. In addition, it is well accepted that A-type granites are formed from relatively high-temperature magmas. Calculated zircon saturation temperatures (T_Zr_) for *Group I* volcanic rocks, using the equation of Watson^37^, are in the range of 760–835 °C with a weighted average of 786 °C, whereas *Group II* volcanic rocks have T_Zr_ varying from 850 to 998 °C with a weighted average of 918 °C (Fig. 8d). These observations indicate that the *Group I* and *Group II* volcanic rocks can respectively be classified as highly fractionated I-type and A-type felsic volcanic rocks. Notably, the studied felsic volcanic rocks exhibit depletions in Eu, Ba and Sr, and have high Rb/Ba and K/Ba ratios, all of which implies that the magmas underwent extensive fractionation of plagioclase and K-feldspar^38,39^. Separation of hornblende may have occurred, as indicated by the strong positive correlation between Dy and Er (Fig. S3a). Moreover, these rocks are depleted in Ti, Nb, Ta, and P, indicating that accessory minerals such as Ti-bearing phases (e.g., rutile, ilmenite, and titanite) and apatite were also fractionated phases. In addition, the (La/Yb)_N_ versus La diagram (Fig. S3b) exhibits that the REE concentrations of the studied volcanic rocks are controlled by the fractionation of allanite, monazite.

#### Origin of I-type felsic volcanic rocks

The I-type felsic volcanic rocks in the HB have high SiO_2_ (70.73–80.35 wt.%) and alkali contents, low concentrations of MgO and transition elements (e.g., Cr, Co and Ni), enrichments in LREEs and LILEs (e.g., Rb, Th and K) and depletions in HREEs and HFSEs (e.g., Nb, Ta and Ti), indicating that the primary magmas were derived from partial melting of crustal material^40–42^. The geochemical diversity of crust-derived magmas is controlled mainly by source compositions and the conditions of melting^38,43^. As discussed above, not only plagioclase but also abundant K-feldspar were fractionated phases^43,44^. This suggests in turn that the magma source, rather than fractional crystallisation, was the dominant mechanism leading to K_2_O enrichment. Therefore, the high-K calc-alkaline felsic volcanic rocks did not result from dehydration melting of tholeiitic amphibolites, which would have produced melts of intermediate to silicic compositions with low K_2_O contents and high Na_2_O/K_2_O ratios^42,45^. Experimental studies indicate that partial melting of tonalitic or granodioritic rocks could generate high-K granitic melts^46^. However, the melts from such a protolith would have similar geochemical characteristics to A-type granites, in sharp contrast to the geochemistry of I-type granites. Thus, tonalitic or granodioritic sources are unlikely.

Sisson^47^ used moderately hydrous medium- to high-K basaltic rocks as starting materials in dehydration melting experiments to produce K-rich granitic–rhyolitic melts that are metaluminous to slightly peraluminous, similar in composition to the felsic volcanic rocks in the HB. Furthermore, the volcanic rocks of the HB have relatively low HFSE and HREE concentrations and zircon saturation temperatures (average 786 °C), and high Rb/Sr ratios, indicating that they could have been derived from a hydrous source in the presence of mica^34,44^. The relatively low Y, Yb and Sr concentrations of the volcanic rocks, coupled with their concave-upwards REE patterns between the middle and heavy REEs, probably record the partial melting of a mafic crust with hornblende and plagioclase as major residual phases^48,49^. If melting occurred with hornblende as a major residual phase, P–T conditions of 10–12.5 kbar and 800–950 °C (corresponding to depths of 30–40 km) are indicated^50^. Taking into account their positive ε_Hf_(t) values (+4.6 to +12.3) and young T_DM2_ ages (918–401 Ma), we conclude that the high-K calc-alkaline I-type felsic volcanic rocks in the HB were probably generated by the partial melting of a dominantly juvenile mica-bearing, K-rich basaltic lower crust.

#### Origin of A-type felsic volcanic rocks

Various A-type felsic rocks worldwide have been formed from a range of magma sources via different processes^36^, and a number of genetic mechanisms have been proposed: (1) direct differentiation of basaltic magma^51^; (2) partial melting of a variety of crustal rocks such as refractory lower-crust granulite after the extraction of hydrous felsic melts^52^, intracrustal tonalitic to granodioritic source rocks^34,35^, or a dry mafic–intermediate middle–lower crust that was dehydrated but not melt depleted^53–56^; and (3) combined crustal and mantle sources, via magma mixing involving mantle and crustal end-members or crustal assimilation and fractional crystallisation (AFC) of basaltic magma^57,58^.

A-type granites generated by extreme differentiation of mantle-derived basaltic magma would be closely associated with a huge volume of contemporaneous mafic–intermediate rocks. However, in the HB, felsic volcanic rocks occur in greater volume than mafic rocks, and intermediate rocks are relatively scarce^23,25^, casting doubt on the validity of this model. The high SiO_2_ (69.43–75.53 wt.%) contents and low MgO (0.17–0.80 wt.%), Cr (0.52–17.71 ppm), Co (0.107–2.65 ppm), and Ni (0.08–7.57 ppm) contents of our A-type felsic volcanic samples indicate a negligible contribution from mantle-derived magmas during their generation. Furthermore, there is no evidence for a mechanism involving magma mixing between mantle-derived and crustal magmas, such as the presence of mafic xenoliths/enclaves, mingling textures, and bimodal or scattered distributions of zircon ε_Hf_(t) values. Therefore, partial melting of a crustal source is the most feasible explanation for the formation of the A-type felsic volcanic rocks in the Hailar Basin.

The high (Na_2_O + K_2_O)/Al_2_O_3_ and TiO_2_/MgO ratios of our A-type felsic volcanic samples are inconsistent with their generation by partial melting of a chemically-depleted residual granulitic source^46,59^. Moreover, the absence of any reports of depleted granulitic terrains in the area that show anatexis with the generation of A-type felsic liquids is also inconsistent with the residual source model^56^. Experimental studies have shown that A-type granitic melts can be produced by the partial melting of crustal igneous rocks of tonalitic to granodioritic composition at low pressures (P ≤ 4 kbar, depths of ≤ 15 km)^34,46^. However, such melts are metaluminous to weakly peraluminous, which is in contrast to the high values of A/CNK (>1) of the peraluminous A-type felsic volcanic rocks in the HB. The prominent negative Eu and Sr anomalies, coupled with high HSFE and HREE concentrations in the A-type felsic volcanic rocks, indicate derivation from a relatively dry and pyroxene-rich source under pressures of 0.8–1.0 GPa^42–60^. Moreover, the A-type felsic volcanic rocks have relatively low Rb/Ba and high K/Rb ratios, which indicates that the K enrichment resulted from the melting of K-feldspar rather than biotite^55^. All of these geochemical characteristics are indicative of a dry, K-rich mafic–intermediate lower crustal resource that was dehydrated but not melt depleted^53^. During the production of the larger-scale I-type magmas from K-rich source rocks, a few K-rich rocks will undergo dehydration accompanied by loss of fluid (primarily H_2_O), but with little or no melt generation^53^. Crustal extension and mantle-derived magmas underplating a dehydrated middle–lower crust would have triggered partial melting, thus producing extensive high-temperature, water-deficient, A-type felsic volcanic rocks in the HB. The generation of peraluminous A-type felsic rocks in this way, by the partial melting of a dehydrated but not melt-depleted mafic–intermediate middle–lower crust, has been demonstrated in previous studies^53–56^. For instance, Jiang^54^ proposed that peraluminous A-type felsic rocks from the Xiangshan volcanic complex of SE China were probably generated by the high-temperature partial melting of metamorphic lower crustal rocks that had been dehydrated during an earlier thermal event.

### Geodynamic implications

The late Mesozoic is regarded as a key period in the transitional tectonics of NE China^7–9^. After the final closure of the Paleo-Asian Ocean at the end of the Permian and beginning of the Early Triassic, the tectonic evolution of NE China during the late Mesozoic was controlled by the Paleo-Pacific and Mongol–Okhotsk tectonic regimes^3,8,10,15,61^. Following the intracontinental contractional deformation and crustal thickening, rift basins such as those at Songliao, Erlian, and Hailar began to develop during the Late Jurassic and Early Cretaceous. This was generally accompanied by voluminous igneous activity and the exhumation of metamorphic core complexes^8,11,29^. However, the deep geodynamic processes that controlled the transformation from compression to extension in NE China remain controversial due to the complexity created by the interfering effects of multiple plates.

**Figure 9:**
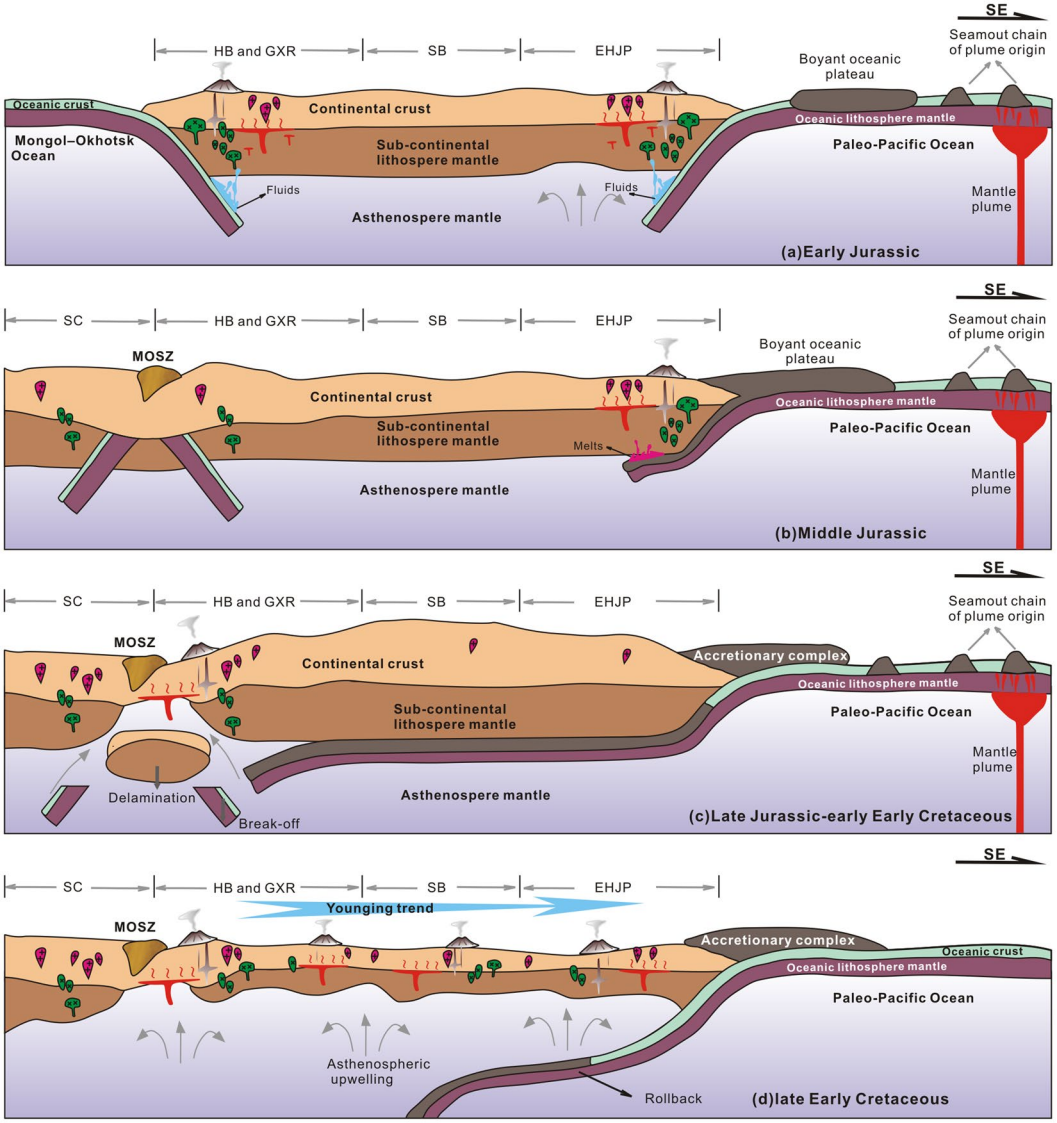
Schematic diagrams showing the geodynamic evolution of NE China during the Mesozoic. Abbreviations shown on the figure are MOSZ = Mongol–Okhotsk Suture Belt, SC = Siberia Craton, HB = Hailar Basin, GXR = Great Xing’an Range, SB = Songliao Basin, and EHJP = Eastern Heilongjiang and Jilin provinces.

To discuss the geodynamic processes of NE China during the late Mesozoic, it is important to understand the spatial extent of the Paleo-Pacific tectonic regime. Early Jurassic mafic intrusions, I-type granitic rocks and calc-alkaline volcanic rocks make up a N–S trending magmatic arc in EHJP and this arc records the magmatic activity caused by the subduction of the Paleo-Pacifc Plate beneath the eastern margin of the Asia continent^8,62,63^. A belt of contemporaneous mafic intrusions and I-type granitic rocks in the LXZR records a bimodal magmatic event that took place during back-arc extension related to the subduction of the Paleo-Pacifc Plate^7,8,64^. These magmatic developments indicate that the subduction angle of the Paleo-Pacifc Plate was steep and that the influence of the Paleo-Pacific subduction was limited at this stage to west of the SB (Fig. 9a). It is worth noting that a striking lull in magmatic activity from the Late Jurassic to the early Early Cretaceous is recorded in the SB, LXZR, and EHJP^8^ (Fig. 7). The best explanation of this lull is flat subduction of the Paleo-Pacifc Plate. The initiation of flat subduction can change the nature of magmatic activity from fluid-related calc-alkaline magmatism typical of a normal magmatic arc to melt-related magmatism (adakite–high-Mg andesite–Nb-enriched basalt)^65^. Once flat subduction has occurred for several million years, the asthenospheric wedge disappears, and a magmatic gap results^66,67^. The occurrence of ca. 174 Ma melt-related volcanic rocks (i.e., high-Mg andesite and Nb-enriched basalt)^63^ and the Late Jurassic–early Early Cretaceous magmatic gap^8^ are probably indicative of the flat subduction of the Paleo-Pacific Plate.

Flat subduction occurs at 10% of present-day convergent margins, and most flat subduction is related to the subduction of aseismic ridges or oceanic plateaux^66^, such as the Inca Plateau in Peru, the Carnegie Ridge in Ecuador and the Euripik Ridge in New Guinea^65,66,68^. In the Paleo-Pacific Ocean, oceanic islands, seamounts and plateaux were formed over a long period from the Carboniferous to the late Mesozoic as a result of plume-related oceanic magmatism^12^. As a result of the Jurassic subduction of the Paleo-Pacific Plate, the seamount chains and plateaux were accreted to the East Asian active margin and incorporated into accretionary complexes such as the Yuejinshan and Raohe accretion complexes of NE China^13^, the Samarka and Tuakha accretionary complexes of the Russian Far East^14^, and the Mino–Tamba and Chichibu accretionary complexes of Japan^12^. When the seamount chains and oceanic plateaux were subducted, the subduction style of the Paleo-Pacific Plate gradually changed from steep to flat because of the high buoyancy of the plate relative to normal oceanic crust^69,70^ (Fig. 9b). The continued flat subduction of the Paleo-Pacific Plate would have resulted in extensive contractional deformation, crustal thickening, and inland migration of the granitoids within NE China, thus building a Middle Jurassic high-standing plateau^10,16,71^, and it would then have led to a magmatic lull in the Late Jurassic–early Early Cretaceous (Fig. 9c). Li^72^ used a flat subduction model to explain the magmatic gap and migration of the Indosinian orogenic front by more than 1300 km from the coast into the continental interior of southeastern China. Thus, it is plausible that the flat subduction of the Paleo-Pacific Plate extended its influence as far as the HB. This interpretation is supported by the later eastward migration of Early Cretaceous volcanism across all of NE China.

In contrast to the Late Jurassic–early Early Cretaceous magmatic quiescence in the coastal region, coeval I- and A-type felsic volcanic rocks and granitoids were developed extensively in the HB and the GXR. These observations indicate that the magmatsm was related to closure of the MOO^8,18,72^. The ca. 168 Ma S-type muscovite granites in the Sunwu area and the 170–160 Ma metamorphic complexes in the Greater and Lesser Xing’an ranges indicate that the MOO to the northwest of the Erguna Massif was closed during the Middle Jurassic^8,73,74^. Break-off of the Mongol–Okhotsk oceanic slab, in response to the final amalgamation of the Siberian Craton and the Mongolian microcontinent during the Late Jurassic, prevented further oceanic subduction owing to the loss of slab pull, and triggered the asthenosphere upwelling and stretching of the lithosphere^16^. Meanwhile, deep-sourced trapped slabs from the Paleo-Asian Ocean would have released water to refertilise the lower crust^15^, thus leading to water-fluxed melting and production of the high-K calc-alkaline I-type felsic volcanic rocks with low zircon saturation temperatures in the HB. At the same time, rocks heated above the I-type magma source would have been dehydrated, thus generating little or no melt during this thermal event. Subsequently, crustal extension and large-scale magma underplating induced by the gravitational collapse of the orogenically thickened crust resulted in partial melting of the dehydrated middle–lower crustal material to produce the high-temperature, water-deficient, A-type felsic volcanic rocks (Fig. 9c). This A-type magmatism was accompanied by the development of extensional basins, such as the HB. The Early Cretaceous volcanic rocks in NE China exhibit an oceanward younging trend, indicating the domains were controlled by the rollback of the Paleo-Pacific Plate (a transition from flat- to normal-angle subduction). In response to slab rollback, magmatism occurred throughout NE China, forming widespread I- and A-type igneous rocks during the Early Cretaceous (Fig. 9d).

## Conclusions

1. New zircon U–Pb dating results indicate that the extensive felsic volcanic rocks of the HB were formed during the Late Jurassic–Early Cretaceous (161–117 Ma).
2. The I-type felsic volcanic rocks formed from magmas derived from partial melting of a dominantly juvenile mica-bearing, K-rich basaltic lower crust, whereas the primary magmas that formed the A-type felsic volcanic rocks were produced during partial melting of a dry mafic–intermediate middle–lower crust that was dehydrated but not melt depleted.
3. The Late Jurassic I- and A-type felsic volcanic rocks in the HB were formed in a post-collisional environment related to break-off of the subducted Mongol–Okhotsk oceanic slab and subsequent gravitational collapse of the orogenically thickened crust after closure of the ocean.
4. The Early Cretaceous I- and A-type felsic volcanic rocks developed in an extensional setting related to rollback of the subducted Paleo-Pacific Plate.

## Methods

### Zircon U–Pb geochronology

Zircons were extracted from whole-rock samples using conventional techniques before handpicking under a binocular microscope. The hand-picked zircons were cast into an epoxy disk and polished to half of their initial thicknesses. Reflected and transmitted light photomicrographs as well as cathodoluminescence (CL) images were obtained to reveal the internal complexities of the zircons and to guide isotopic analysis. Zircon U–Pb analyses were performed using an Agilent 7500a inductively coupled plasma–mass spectrometer (ICP–MS) equipped with a 193 nm laser (New Wave Research), housed at the IGGCAS, Beijing, China. Zircon 91500 was used as the external standard for isotope fractionation. The zircon standard TEMORA (417 Ma)^75^ was used as a secondary standard when examining the deviations in age measurements and calculations. Procedural details were shown in Xie^76^. Isotopic ratios and element concentrations were calculated using the Glitter (version 4.4, Macquarie University) program. The data were processed using the Isoplot program of Ludwig^77^.

### Major and trace element

The samples for major and trace element analyses were trimmed to remove weathered surfaces, cleaned with de-ionised water, crushed, and ground to ~200 mesh in a specially designed mill. Major element concentrations were determined using an ICP–optical emission spectroscope (ICP–OES; Leeman Prodigy) at the Geological Laboratory Centre, China University of Geosciences, Beijing, China. Based on the United States Geological Survey (USGS) rock standard AGV-2 and the Chinese National Geological Reference (CNGR) material GSR-1, the analytical uncertainties were generally <1% for most major element oxides with the exception of TiO_2_ (~1.5%) and P_2_O_5_ (~2.0%). Trace elements were analysed using an ICP–MS. Rock standards AGV-2 and GSR-1 were used for analytical quality control. For further details of the analytical procedures, see Wang^78^.

### Zircon Hf isotopes

*In situ* zircon Hf isotope analyses were undertaken using a Neptune multi-collector ICP–MS with a Newwave 193 nm laser system at the Tianjin Institute of Geology and Mineral Resources, Tianjin, China. Instrument settings and a detailed outline of the analytical procedures are given by Geng^79^. The Hf analyses were measured on the dated spots as the U–Pb isotope analyses, with the diameter of a laser spot size of 50 μm and the ablation rate of 8 Hz. Zircon GJ-1 was used as the reference standard, giving a weighted mean ^176^Hf/^177^Hf ratio of 0.282020 ± 0.000008 (2σ, n = 46), which is in accord with the weighted mean ^176^Hf/^177^Hf ratio of 0.282000 ± 0.000005 (2σ) measured by the solution analysis method^80^. Measured ^176^Hf/^177^Hf and ^176^Lu/^177^Hf ratios were used to calculate initial ^176^Hf/^177^Hf ratios, employing a decay constant of 1.865 × 10^–11^ yr^–1^ for ^176^Lu^81^. The calculation of εHf(t) values was based on the present-day chondritic ratios of ^176^Hf/^177^Hf = 0.282772 and ^176^Lu/^177^Hf = 0.0332^82^. The calculation of the two-stage Hf model (TDM2) ages was based on an average crustal value (f_cc_) of –0.548^83^.

## Supplementary information

Supplementary Information
