# Supplementary material for: Generation of late Mesozoic felsic volcanic rocks in the Hailar Basin, northeastern China in response to overprinting of multiple tectonic regimes
Source: Sci Rep. 2019 Nov 1;9:15854. doi: 10.1038/s41598-019-52181-x (PMC6825236; doi:10.1038/s41598-019-52181-x)
Supplement: Supplementary file 1 — Supplementary Information [file 41598_2019_52181_MOESM1_ESM.pdf]

Generation of late Mesozoic felsic volcanic rocks in the Hailar Basin, northeastern China in response to overprinting of multiple tectonic regimes

Zheng Ji [a], Qi-An Meng [b], Chuan-Biao Wan [b], De-Feng Zhu [b], Wen-Chun Ge [a,*], Yan-Long Zhang [a], Hao Yang [a], Yu Dong [a], Yan Jing [a]

[a] *College of Earth Sciences, Jilin University, Changchun 130061, China*

[b] *Exploration and Development Research Institute, Daqing Oilfield Limited Company, Daqing 163712, China*

*Corresponding author: College of Earth Sciences

Jilin University

No. 2199 Jianshe Street

Changchun 130061, China

Tel: 86-0431-88502278

Fax: 86-0431-88584422

E-mail: gewenchun@jlu.edu.cn

Online supplementary information

Includes the following materials:

1. Supplementary figures.

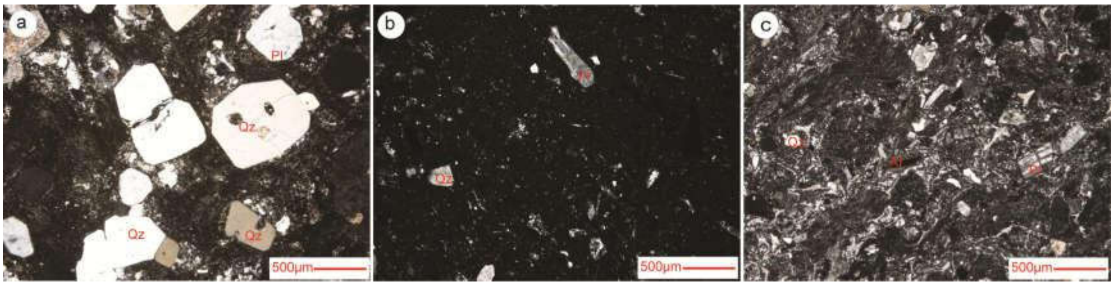

Figure S1. Representative photomicrographs (cross-polarised light) of late Mesozoic felsic volcanic rocks in the Hailar Basin. (a) Rhyolite (Sample C4-2). (b) Dacite (Sample Bei39-1). (c) Rhyolitic tuff (Sample 08GW080).

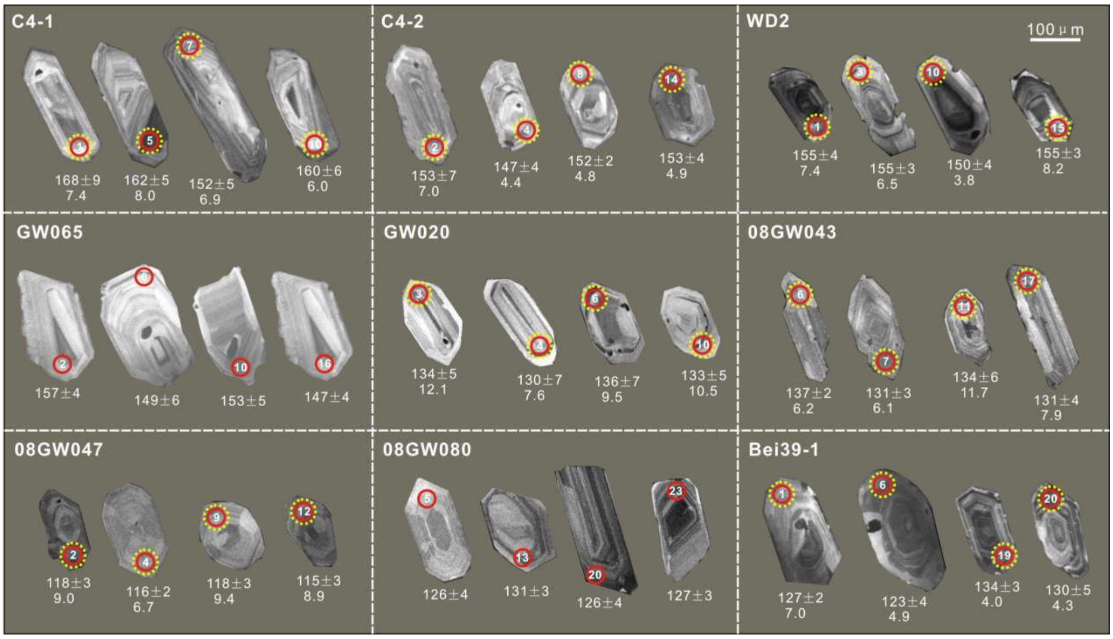

Figure S2. Cathodoluminescence (CL) images of representative zircons from the late Mesozoic felsic volcanic rocks in the Hailar Basin. U–Pb analytical spots are indicated by small solid circles and Lu–Hf analytical spots by large dotted circles.

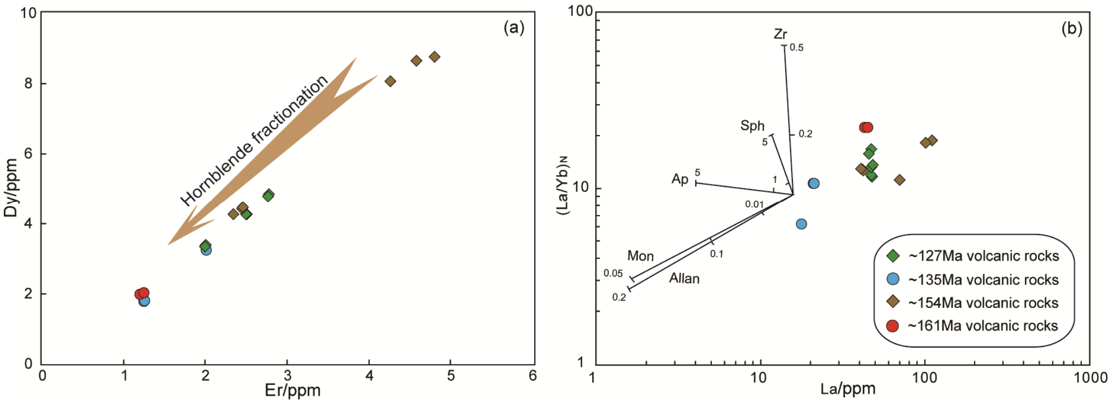

Figure S3. Plots of (b) Dy versus Er and (b) $(La/Yb)_N$ versus La for late Mesozoic felsic volcanic rocks in the Hailar Basin. Ap = apatite; Zr = zircon; Allan = allanite; Mon = monazite; Sph = sphene.

2. Supplementary tables

Table S1. Zircon U–Pb ages for late Mesozoic volcanic rocks in NE China.

Order	Sample	Location	Latitude	Longitude	Formation	Lithology	Age (Ma)	References
Hailar Basin and Great Xing'an Range								
1	GW03285	Mangui	52°03′ 27″ N	122°05′ 33″ E	Dyke	Dolerite	133±3	Ref[1]
2	9411-A	Zhalantun	48°00′ 12″ N	122°46′ 19″ E	Dyke	Granite porphyry	130±1	Ref[1]
3	GW04005	Zhalantun	48°00′ 12″ N	122°46′ 19″ E	Dyke	Coarse dolerite	124±2	Ref[1]
4	FW04-420	Langdonggou	48°16′ 31″ N	123°38′ 12″ E	Yilikede	Basaltic andesite	123±2	Ref[1]
5	GW03120	Walagan	52°39′ 39″ N	124°19′ 42″ E	Yilikede	Basalt	126±5	Ref[1]
6	GW04027	GW04027	48°51′ 11″ N	121°37′ 27″ E	Yilikede	Basalt	112±2	Ref[1]
7	GW04032	Mianduhe	49°07′ 01″ N	120°55′ 43″ E	Yilikede	Basalt	117±1	Ref[1]
8	FW04-401	Nuomin	49°14′ 31″ N	123°46′ 04″ E	Shangkuli	Andesite	123±1	Ref[1]
9	FW04-409	Yilizhen	49°34′ 00″ N	124°14′ 18″ E	Shangkuli	Rhyolite	118±2	Ref[1]
10	FW04-418	Huoerqi	48°23′ 02″ N	123°19′ 28″ E	Shangkuli	Dacite	113±2	Ref[1]
11	FW04-419	FW04-419	48°15′ 45″ N	123°45′ 47″ E	Shangkuli	Dacite	116±1	Ref[1]
12	GW04029	Gaojishanlinchang	48°48′ 05″ N	121°09′ 32″ E	Shangkuli	Rhyolite	119±2	Ref[1]
13	GW04034	Mianduhe	49°07′ 15″ N	120°54′ 22″ E	Shangkuli	Rhyolite	111±1	Ref[1]
14	GW04036	Shangkuli	50°14′ 47″ N	120°28′ 08″ E	Shangkuli	Rhyolite	119±2	Ref[1]
15	GW04044	Niuerhe	51°32′ 04″ N	122°01′ 08″ E	Shangkuli	Rhyolite	135±1	Ref[1]
16	GW03148	Pangu	52°38′ 27″ N	124°41′ 09″ E	Shangkuli	Rhyolite	128±1	Ref[1]
17	GW03155	Pangu	52°38′ 27″ N	124°41′ 09″ E	Shangkuli	Rhyolite	125±1	Ref[1]
18	GW04037	Sanhezhen	50°26′ 04″ N	120°08′ 59″ E	Tamulangou	Olivine basalt	139±2	Ref[1]
19	9434	Boketu	48°44′ 12″ N	121°55′ 20″ E	Tamulangou	Rhyolitic tuff	139±1	Ref[1]
20	9422	Balin Station	48°17′ 57″ N	122°19′ 09″ E	Tamulangou	Rhyolitic tuff	141±4	Ref[1]
21	GW04010	Nanmu	48°13′ 01″ N	122°20′ 37″ E	Tamulangou	Rhyolitic tuff	146±2	Ref[1]
22	GW04011	Balin Station	48°17′ 57″ N	122°19′ 09″ E	Tamulangou	Rhyolitic tuff	148±2	Ref[1]
23	GW04257	Tamulangou	48°09′ 13″ N	121°14′ 44″ E	Tamulangou	Basalt	128±8	Ref[1]
24	GW04042	Tamulangou	51°25′ 56″ N	121°31′ 52″ E	Tamulangou	Basalt	186±3	Ref[1]
25	07ZH017	Alider	46°15′ 16″ N	121°05′ 34″ E	Manketouebo	Dacite	138±1	Ref[2]
26	06ZH049	Balimuhade	44°55′ 26″ N	120°32′ 43″ E	Manitu	Andesite	132±1	Ref[2]
27	06ZH043	Aletandaban	45°07′ 32″ N	120°01′ 08″ E	Baiyingaolao	Rhyolite	129±1	Ref[2]
28	07ZH065	Jiefangtun	45°38′ 23″ N	121°10′ 50″ E	Meiletu	Basaltic andesite	126±4	Ref[2]
29	05FW060	Maoshandong	43°00′ 13″ N	118°24′ 03″ E	Manketouebo	Rhyolite	174±4	Ref[2]
30	06ZH032	Baritu	44°53′ 11″ N	119°23′ 40″ E	Manketouebo	Rhyolite	133±2	Ref[2]
31	06ZH110	Paotai	43°02′ 23″ N	118°21′ 04″ E	Manketouebo	Rhyolite	154±1	Ref[2]
32	07ZH097	Huolinhe	45°36′ 08″ N	119°33′ 11″ E	Manketouebo	Rhyolite	150±2	Ref[2]
33	07ZH110	Manketouebo	45°14′ 08″ N	119°53′ 52″ E	Manketouebo	Rhyolite	156±1	Ref[2]
34	II06029-1	Manketouebo	45°13′ 56″ N	119°54′ 45″ E	Manketouebo	Rhyolite	153±8	Ref[2]
35	07ZH172	Manitu	44°04′ 54″ N	118°45′ 50″ E	Manketouebo	Rhyolite	163±1	Ref[2]
36	II06031-2	Manitu	44°05′ 36″ N	118°47′ 29″ E	Manketouebo	Rhyolite	160±2	Ref[2]
37	II06032-1	Manitu	44°05′ 36″ N	118°47′ 29″ E	Manketouebo	Rhyolite	165±1	Ref[2]
38	07ZH155	Sebuer	45°05′ 45″ N	120°44′ 11″ E	Manketouebo	Dacite	151±1	Ref[2]
39	GW04199	Sancha	46°39′ 40″ N	120°08′ 27″ E	Manketouebo	Dacite	124±2	Ref[2]
40	07ZH104	Huolinhe	45°32′ 44″ N	119°31′ 45″ E	Manketouebo	Andesite	157±2	Ref[2]
41	07ZH140	Gahaitu	44°56′ 36″ N	121°01′ 30″ E	Manketouebo	Andesite	150±3	Ref[2]

42	07ZH086	Tuliemaodu	45°33′ 26″ N 120°53′ 45″ E	Manketouebo	Rhyolitic tuff	128±1	Ref[2]
43	07ZH208	Shanghuofang	43°06′ 34″ N 117°40′ 35″ E	Manketouebo	Ignimbrite	135±2	Ref[2]
44	07ZH224	Tuchengzi	43°01′ 00″ N 118°22′ 52″ E	Manketouebo	Ignimbrite	153±2	Ref[2]
45	07ZH006	Baoantun	46°08′ 52″ N 121°17′ 31″ E	Manitu	Andesite	151±2	Ref[2]
46	07ZH181	Manitu	44°04′ 59″ N 118°45′ 37″ E	Manitu	Andesite	138±1	Ref[2]
47	G0221-2	Keyouzhongqi	44°59′ 27″ N 121°17′ 02″ E	Manitu	Andesite	129±2	Ref[2]
48	GW04136	Eti	45°55′ 44″ N 121°46′ 40″ E	Manitu	Andesite	125±1	Ref[2]
49	GW04137	Naoniushan	45°47′ 16″ N 121°40′ 36″ E	Manitu	Andesite	127±3	Ref[2]
50	07ZH236	Paotai	43°04′ 03″ N 118°36′ 02″ E	Manitu	Dacite	156±1	Ref[2]
51	07ZH193	Wulahai	43°41′ 07″ N 118°42′ 43″ E	Manitu	Rhyolite	155±1	Ref[2]
52	07ZH081	Herimu	45°34′ 15″ N 120°53′ 18″ E	Manitu	Rhyolitic tuff	126±1	Ref[2]
53	GW04185	Sharentai	46°21′ 18″ N 120°34′ 21″ E	Baiyingaolao	Rhyolite	135±1	Ref[2]
54	GW04215	Arshan	47°09′ 07″ N 119°56′ 33″ E	Baiyingaolao	Rhyolite	124±1	Ref[2]
55	06ZH015	Huitonghe	44°16′ 44″ N 118°35′ 29″ E	Baiyingaolao	Rhyolite	131±1	Ref[2]
56	06ZH055	Bayartuhushuo	45°05′ 32″ N 120°22′ 38″ E	Baiyingaolao	Felsite	132±1	Ref[2]
57	07ZH078	Wulanhade	45°39′ 20″ N 120°47′ 39″ E	Baiyingaolao	Rhyolite	131±1	Ref[2]
58	07ZH012	Zhongxintun	46°07′ 56″ N 121°10′ 23″ E	Baiyingaolao	Ignimbrite	137±1	Ref[2]
59	07ZH170	Baiyingaolao	43°55′ 59″ N 119°17′ 41″ E	Baiyingaolao	Ignimbrite	138±1	Ref[2]
60	07ZH212	Shanghuofang	43°07′ 54″ N 117°41′ 37″ E	Baiyingaolao	Ignimbrite	139±2	Ref[2]
61	07ZH028	Baoshi	46°02′ 44″ N 121°07′ 21″ E	Meiletu	Andesite	128±9	Ref[2]
62	07ZH142	Chaganbulage	45°00′ 23″ N 120°55′ 22″ E	Meiletu	Andesite	128±1	Ref[2]
63	07ZH072	Zhangjiajie	45°52′ 09″ N 120°52′ 42″ E	Meiletu	Andesite	128±2	Ref[2]
64	07ZH116	Wulanhada	45°17′ 05″ N 120°34′ 37″ E	Meiletu	Andesite	134±2	Ref[2]
65	06ZH036	Meiletu	45°12′ 54″ N 119°32′ 42″ E	Meiletu	Andesite	131±1	Ref[2]
66	06ZH060	Wulanhada	45°15′ 39″ N 120°34′ 23″ E	Meiletu	Andesite	128±1	Ref[2]
67	LD04-1	Lindong	43°55′ 45″ N 119°26′ 47″ E	Manketouebo	Rhyolite	120±5	Ref[3]
68	DBS04-1	Daban	44°02′ 00″ N 118°48′ 42″ E	Manketouebo	Dacite	133±1	Ref[3]
69	TQBS04-2	Tuquan	46°02′ 40″ N 121°07′ 22″ E	Manitu	Andesite	140±1	Ref[3]
70	LDDS04-2	Lindong	44°10′ 57″ N 119°16′ 56″ E	Manitu	Rhyolite	139±2	Ref[3]
71	ZLTXS04-1	Zalute	44°29′ 28″ N 120°34′ 06″ E	Manitu	Rhyolite	125±1	Ref[3]
72	LDDS04-1	Lindong	44°10′ 57″ N 119°15′ 37″ E	Baiyingaolao	Rhyolite	125±4	Ref[3]
73	ZLTHJ04-3	Zalute	44°45′ 16″ N 121°07′ 44″ E	Meiletu	Basaltic andesite	122±2	Ref[3]
74	TQBS04-3	Tuquan	45°41′ 54″ N 121°16′ 26″ E	Manketouebo	Rhyolite	152±2	Ref[3]
75	GW034	Tu#1	49°29′ 30″ N 117°53′ 19″ E	Shangkuli	Rhyolitic tuff	119±2	Ref[4]
76	GW035	Tu#1	49°29′ 30″ N 117°53′ 19″ E	Shangkuli	Rhyolitic tuff	117±1	Ref[4]
77	GW036	Tu#1	49°29′ 30″ N 117°53′ 19″ E	Tamulantou	Trachyandesite	151±6	Ref[4]
78	08GW032	Hong#2	48°57′ 42″ N 118°17′ 50″ E	Budate	Rhyolite	145±2	Ref[4]
79	08GW033	Hong#2	48°57′ 42″ N 118°17′ 50″ E	Budate	Rhyolite	146±2	Ref[4]
80	H2-1837	Hong#2	48°57′ 42″ N 118°17′ 50″ E	Budate	Rhyolite	145±2	Ref[4]
81	H2-2032	Hong#2	48°57′ 42″ N 118°17′ 50″ E	Budate	Rhyolite	149±3	Ref[4]
82	08GW036	Hong#2	48°57′ 42″ N 118°17′ 50″ E	Budate	Rhyolite	149±2	Ref[4]
83	MZ2-1	Western Hulun Lake	49°17′ 44″ N 117°31′ 30″ E	Shangkuzi	Andesite	166±2	Ref[5]
84	MZ5-3	Western Hulun Lake	49°20′ 06″ N 117°30′ 33″ E	Shangkuli	Dacite	125±1	Ref[5]
85	MZ20-1	Dashimo	49°25′ 27″ N 117°04′ 48″ E	Shangkuli	Trachydacite	127±3	Ref[5]

86	MZ10-1	Hulun Lake	49°23′ 56″ N 117°25′ 21″ E	Shangkuli	Pyroxene andesite	125±2	Ref[5]
87	ZKX24-04	Well depth of 500m	49°21′ 36″ N 117°33′ 28″ E	Shangkuli	Trachydacite	142±1	Ref[5]
88	MZ1-1	Hulun Lake	49°17′ 33″ N 117°31′ 36″ E	Shangkuli	Dacitic ignimbrite	141±1	Ref[5]
89	MZ21-1	Dashimo	49°26′ 42″ N 117°02′ 31″ E	Tamulantou	Olivne basalt	129±2	Ref[5]
90	ZKX24-00	Well depth of 255m	49°19′ 16″ N 117°32′ 22″ E	Shangkuli	Basaltic andesite	123±2	Ref[5]
91	MZ7-1	Western Hulun Lake	49°21′ 16″ N 117°34′ 22″ E	Shangkuli	Andesite	125±2	Ref[5]
92	ER17-1	Eastern Ehe town	50°44′ 45″ N 120°11′ 19″ E	Shangkuli	Rhyolite	143±4	Ref[6]
93	ER3-1	Genheqiaobei	50°19′ 57″ N 120°15′ 01″ E	Tamulangou	Basaltic andesite	125±2	Ref[6]
94	ER19-2	Shanghulin	50°42′ 37″ N 120°12′ 52″ E	Tamulangou	Basaltic andesite	127±1	Ref[6]
95	ZKD2-1	Shanghulin	50°46′ 32″ N 120°11′ 35″ E	Tamulangou	Basaltic andesite	128±3	Ref[6]
96	ER16-1	Eastern Ehe town	50°45′ 57″ N 120°10′ 37″ E	Tamulantou	Rhyolite	124±1	Ref[6]
97	ER1-1	Genhe	49°59′ 57″ N 120°06′ 50″ E	Meiletu	Trachyandesite	128±2	Ref[6]
98	ER9-1	Eastern Ehe town	50°47′ 12″ N 119°52′ 54″ E	Shangkuli	Trachydacite	125±1	Ref[6]
99	ER5-1	Genheqiaobei	50°26′ 14″ N 120°00′ 54″ E	Shangkuli	Pyroxene andesite	114±3	Ref[6]
100	M080-1	Daba	48°53′ 24″ N 116°20′ 57″ E	Manitu	Trachyte	156±1	Ref[7]
101	M036-1	Bayangshan	48°45′ 03″ N 116°26′ 29″ E	Manitu	Trachyandesite	158±1	Ref[7]
102	M111-1	Linquan	48°51′ 14″ N 116°58′ 54″ E	Manitu	Trachyandesite	146±1	Ref[7]
103	M003-1	Manzhouli	48°45′ 03″ N 116°26′ 29″ E	Tamulantou	Trachyandesite	161±2	Ref[8]
104	M043-1	Manzhouli	48°53′ 24″ N 116°20′ 57″ E	Tamulantou	Trachyandesite	161±1	Ref[8]
105	M133-1	Manzhouli	49°36′ 11″ N 116°40′ 50″ E	Tamulantou	Trachyandesite	164±1	Ref[8]
106	M054-6	Xinyouqi	48°37′ 14″ N 116°49′ 03″ E	Shangkuli	Rhyolite	141±1	Ref[9]
107	M122-1	Linquan	49°19′ 49″ N 117°29′ 47″ E	Shangkuli	Rhyolite	139±1	Ref[9]
108	M132-1	Matoushan	49°32′ 35″ N E116°44′ 41″	Shangkuli	Rhyolite	141±1	Ref[9]
109	M138-1	Shidamo	49°30′ 08″ N 117°11′ 29″ E	Shangkuli	Rhyolite	141±1	Ref[9]
110	ER18-1	Shanghulin	50°44′ 02″ N 120°11′ 57″ E	Wanbao	Andesite	182±2	Ref[10]
111	M271-16	Southern Manzhouli	48°29′ 05″ N 116°50′ 13″ E	Yilikede	Trachyandesite	135±2	Ref[11]
112	ZKD0042	Southern Manzhouli	49°23′ 49″ N 117°33′ 47″ E	Shangkuli	Rhyolite	142±1	Ref[11]
113	M467-1	Southern Manzhouli	49°06′ 43″ N 117°11′ 52″ E	Jixiangfeng	Rhyolite	156±2	Ref[11]
114	M276-1	Southern Manzhouli	48°29′ 43″ N 116°24′ 58″ E	Jixiangfeng	Rhyolite	161±1	Ref[11]
115	M007-1	Southern Manzhouli	48°16′ 55″ N 117°05′ 35″ E	Jixiangfeng	Rhyolite	162±2	Ref[11]
116	D0570-5-1	Wuchagou	46°57′ 40″ N 120°22′ 04″ E	Baiyingaolao	Rhyolitic tuff	134±1	Ref[12]
117	D9805	Wuchagou	46°48′ 12″ N 120°22′ 11″ E	Baiyingaolao	Rhyolitic tuff	133±1	Ref[12]
118	D9806-2	Wuchagou	46°48′ 36″ N 120°22′ 3″ E	Baiyingaolao	Rhyolite	133±1	Ref[12]
119	PM002-18-1	Wuchagou	46°46′ 38″ N 120°17′ 49″ E	Baiyingaolao	Rhyolitic tuff	130±3	Ref[12]
120	D9951	Wuchagou	46°46′ 50″ N 120°17′ 07″ E	Baiyingaolao	Rhyolitic tuff	132±2	Ref[12]
121	TW2	Tuliemaodu	45°37′ 41″ N 120°54′ 06″ E	Meiletu	Andesite	127±2	Ref[13]
122	TW1	Tuliemaodu	45°31′ 10″ N 120°44′ 11″ E	Baiyingaolao	Rhyolite	127±2	Ref[13]
123	TW8	Tuquan	45°35′ 19″ N 121°20′ 38″ E	Manitu	Andesite	127±3	Ref[13]
124	TW3	Tuliemaodu	45°35′ 17″ N 120°55′ 23″ E	Manitu	Andesite	130±2	Ref[13]
125	TW4	Tuquan	45°32′ 19″ N 121°10′ 57″ E	Manitu	Trachyandacite	138±2	Ref[13]
126	TW9	Tuquan	45°37′ 57″ N 121°22′ 54″ E	Manitu	Trachyandacite	142±3	Ref[13]
127	TW6	Tuquan	45°35′ 45″ N 121°15′ 10″ E	Manketouebo	Dacite	147±2	Ref[13]
128	TW7	Tuquan	45°38′ 42″ N 121°15′ 30″ E	Manketouebo	Dacite	154±2	Ref[13]

129	ER18-1	Shanghulin	50°44′02″N	120°11′57″E	Tamulantou	Trachybasalt	182±2	Ref[13]
130	13ER20-1	Mordaoga	51°23′27″N	121°03′07″E	Tamulantou	Basaltic andesite	193±5	Ref[13]
131	13ER52-1	Taohai	49°32′33″N	119°51′11″E	Tamulantou	Basaltic andesite	181±9	Ref[13]
132	D0186-1-1	Wuchagou	46°47′07″N	120°32′27″E	Manketouebo	Rhyolitic tuff	140±1	Ref[14]
133	PM005-1-1	Wuchagou	46°43′48″N	120°35′31″E	Manketouebo	Rhyolitic tuff	143±2	Ref[14]
134	PM005-6-1	Wuchagou	46°44′10″N	120°35′24″E	Manketouebo	Rhyolitic tuff	139±1	Ref[14]
135	PM012-1-1	Wuchagou	46°56′01″N	120°43′10″E	Manketouebo	Rhyolite	141±1	Ref[14]
136	D5104	Dayangshu	49°04′11″N	124°04′20″E	Ganhe	Trachyandesite	109±2	Ref[15]
137	D5428-1	Dayangshu	49°05′43″N	123°55′41″E	Ganhe	Trachyandesite	114±2	Ref[15]
138	D9832-1	Wuchagou	46°50′30″N	120°40′35″E	Xinmin	Rhyolite	162±2	Ref[16]
139	D9843-1	Wuchagou	46°51′12″N	120°29′45″E	Xinmin	Rhyolite	154±1	Ref[16]
140	D9845-1	Wuchagou	46°51′54″N	120°28′44″E	Xinmin	Rhyolite	157±1	Ref[16]
141	D9849-1	Wuchagou	46°50′7″N	120°25′19″E	Xinmin	Rhyolite	161±1	Ref[16]
142	08GW021	Chu#2			Nantun	Rhyolite	128±2	Ref[17]
143	08GW023	Chu#4			Xing'anling	Rhyolite	120±1	Ref[17]
144	08GW024	Chu#4			Xing'anling	Rhyolite	127±4	Ref[17]
145	08GW025	Chu#4			Xing'anling	Rhyolite	125±1	Ref[17]
146	08GW026	Chu#4			Xing'anling	Rhyolite	125±1	Ref[17]
147	08GW027	Chu#4			Xing'anling	Rhyolite	126±1	Ref[17]
148	C4-1	Chu#4			Tamulantou	Rhyolite	161±2	This study
149	C4-2	Chu#4			Tamulantou	Rhyolite	150±2	This study
150	WD2	WD#2			Tamulantou	Rhyolite	156±3	This study
151	GW065	WD#2			Tamulantou	Rhyolite	154±2	This study
152	08GW020	He#9			Tongbomiao	Rhyolite	136±3	This study
153	08GW043	Bei#16			Tongbomiao	Rhyolite	135±2	This study
154	08GW047	Bei#38			Nantun	Rhyolite	118±1	This study
155	08GW080	Bei#20			Nantun	Rhyolite	127±2	This study
156	Bei39-1	Bei#39			Nantun	Rhyolite	128±2	This study
Songliao Basin								
1	PK10-6	Shiwu fault	43°53′31″N	124°14′49″E	Huoshiling	Trachydacite	129±1	Ref[18]
2	SN190-12	Western slope	44°55′00″N	123°24′43″E	Huoshiling	Trachydacite	133±1	Ref[18]
3	SN72-5	Shiwu fault	43°40′52″N	124°36′56″E	Yingcheng	Basaltic andesite	118±1	Ref[18]
4	SN56-7	Shiwu fault	43°47′58″N	124°29′05″E	Yingcheng	Trachydacite	116±1	Ref[18]
5	L47-1	Shiwu fault	43°49′11″N	124°28′35″E	Yingcheng	Basaltic andesite	114±4	Ref[18]
6	SN108-2	Central depression	43°40′52″N	124°02′43″E	Yingcheng	Rhyolite	119±1	Ref[18]
7	SN118-1	Shiwu fault	43°50′40″N	124°32′30″E	Yingcheng	Basaltic andesite	110±16	Ref[18]
8	S202	Shengping			Yingcheng	Rhyolite	112±1	Ref[19]
9	X95	Xingcheng			Yingcheng	Rhyolite	113±2	Ref[19]
10	Psc7	Chengzijie			Yingcheng	Rhyolite	116±2	Ref[20]
11	Psc10	Chengzijie			Yingcheng	Rhyolite	115±1	Ref[20]
12	Psc12	Chengzijie			Yingcheng	Rhyolite	117±2	Ref[20]
13	PLT218	Chengzijie			Yingcheng	Rhyolite	116±3	Ref[20]
14	PLT195	Chengzijie			Yingcheng	Rhyolite	110±2	Ref[20]
15	X9a	Xingcheng			Yingcheng	Rhyolite	112±1	Ref[21]

16	X9b	Xingcheng			Yingcheng	Rhyolite	113±1	Ref[21]
17	X201a	Xingcheng			Yingcheng	Rhyolite	111±2	Ref[21]
18	SL0904	Guanma	44°23′ 14″ N	126°08′ 11″ E	Yingcheng	Rhyolite	113±2	Ref[22]
19	SL0919	Guanma	44°23′ 14″ N	126°08′ 11″ E	Yingcheng	Rhyolite	114±2	Ref[22]
20	LS3-1	Lindian			Yingcheng	Dacite	119±4	Ref[23]
21	LS3-2	Lindian			Yingcheng	Dacite	120±2	Ref[23]
22	LS3-3	Lindian			Yingcheng	Dacite	114±10	Ref[23]
23	WZ01	Northen depression			Yingcheng	Rhyolite	114±2	Ref[24]
24	WZ03	Northen depression			Yingcheng	Rhyolite	115±2	Ref[24]
25	WZ09	Northen depression			Yingcheng	Rhyolite	112±2	Ref[24]
26	WZ05	Southeastern depression			Yingcheng	Andesite	109±2	Ref[24]
27	WZ28	Southeastern depression			Yingcheng	Rhyolite	115±2	Ref[24]
28	WZ30	Southeastern depression			Yingcheng	Rhyolite	110±2	Ref[24]
29	SS2-1-10	Shengping			Yingcheng	Rhyolite	113±1	Ref[25]
30	SS2-1-14	Shengping			Yingcheng	Rhyolite	113±1	Ref[25]
31	SS202-4	Shengping			Yingcheng	Rhyolite	112±1	Ref[25]
32	XS601-4	Xingcheng			Yingcheng	Rhyolite	112±2	Ref[25]
33	XS8-1	Xingcheng			Yingcheng	Rhyolite	111±1	Ref[25]
34	XS9-2	Xingcheng			Yingcheng	Rhyolite	112±1	Ref[25]
35	XS9-5	Xingcheng			Yingcheng	Rhyolitc tuff	113±2	Ref[25]
36	XS201-1	Xingcheng			Yingcheng	Rhyolitc tuff	111±1	Ref[25]
37	XS502-2	Xingcheng			Yingcheng	Rhyolitc tuff	115±1	Ref[25]
Eastern Heilongjiang-Jilin provinces								
1	HTW4-1	Northeastern Yichun city	48°44′ 09″ N	129°20′ 05″ E	Ganhe	Andesite	108±1	Ref[10]
2	HYC1-1	Qingshan	47°56′ 37″ N	128°53′ 57″ E	Youhao	Rhyolite	102±1	Ref[10]
3	HB38-1	Heilonggong	45°25′ 30″ N	127°53′ 21″ E	Upper Heilonggong	Basaltic andesite	173±3	Ref[10]
4	HB13-1	Hongxing	45°34′ 07″ N	127°08′ 18″ E	Wudaoling	Basalte	174±2	Ref[10]
5	HB10-1	Sandaogang	45°42′ 25″ N	127°19′ 33″ E	Wudaoling	Rhyolite	175±1	Ref[10]
6	HYC13-1	Sandaogang	47°33′ 47″ N	128°24′ 04″ E	Wudaoling	Rhyolite	185±1	Ref[10]
7	HB28-1	Wangjiaguanzi	45°32′ 20″ N	127°47′ 36″ E	Taiantun	Dacite	190±1	Ref[10]
8	HYC2-1	Qingshan	47°56′ 37″ N	128°53′ 57″ E	Taiantun	Rhyolite	187±2	Ref[10]
9	HB4-1	Shisanhucun	45°35′ 10″ N	127°41′ 46″ E	Ningyuancun	Rhyolite	190±1	Ref[10]
10	HYL2-1	Bayan	46°11′ 55″ N	127°39′ 28″ E	Maoershan	Trachyandesite	179±2	Ref[10]
11	HYL3-1	Bayan	46°11′ 55″ N	127°39′ 30″ E	Maoershan	Rhyolite	184±2	Ref[10]
12	HS8-2	Suiyangzhen	44°25′ 06″ N	130°55′ 18″ E	Shuangqiaozi	Dacite	184±2	Ref[10]
13	HD6-1	Laoheishan	43°38′ 46″ N	130°45′ 54″ E	Luoquanzhan	Basaltic andesite	180±1	Ref[10]
14	HS1-1	Maqiaohe	44°41′ 38″ N	130°33′ 11″ E	Yilin	Dacite	96±3	Ref[10]
15	JRH01	Changzhengqiao	46°31′ 30″ N	133°38′ 39″ E	Pikeshan	Dacite	116±2	Ref[10]
16	JRH03	Quanfa	46°08′ 56″ N	132°50′ 39″ E	Songmuhe	Andesite	129±3	Ref[10]
17	HY1-1	Dongfanghong	46°26′ 51″ N	129°48′ 11″ E	Songmuhe	Andesite	112±1	Ref[10]
18	HY4-1	Qinglongshan	46°27′ 24″ N	130°07′ 25″ E	Songmuhe	Dacite	110±2	Ref[10]
19	HM2-1	Xingkai	45°44′ 27″ N	132°02′ 60″ E	Songmuhe	Rhyolite	122±2	Ref[10]
20	HM1-1	Peide	45°39′ 47″ N	131°52′ 01″ E	Peide	Rhyolite	116±1	Ref[10]

21	HQ3-1	Qitaihe	45°48′ 57″ N 130°57′ 04″ E	Yilin	Rhyolite	124±3	Ref[10]
22	YH10-1	Helong	43°26′ 30″ N 129°00′ 45″ E	Quanshuicun	Andesite	108±3	Ref[10]
23	YB2-12	Wangqiang	43°15′ 25″ N 130°07′ 07″ E	Jingouling	Andesite	107±1	Ref[10]
24	YB1-1	Tianqiaoling	43°20′ 23″ N 129°22′ 54″ E	Tianqiaoling	Dacite	175±3	Ref[10]
25	YW3-12-12	Manhe	43°12′ 24″ N 129°58′ 09″ E	Manhe	Andesite	187±6	Ref[10]
26	YB4-1	Tuntianying	43°07′ 55″ N 129°07′ 54″ E	Tuntianying	Andesite	97±1	Ref[10]
27	YB4-9	Badaogou	43°00′ 18″ N 129°11′ 19″ E	Tuntianying	Dacite	88±2	Ref[10]
28	YB2-1	Ciweigou	43°09′ 50″ N 129°34′ 19″ E	Tuntianying	Andesite	176±3	Ref[10]
29	SF01-1	Suifenhe	44°21′ 39″ N 131°02′ 52″ E	Suifenhe	Dacite	93±1	Ref[26]
30	SF03-4	Suifenhe	44°23′ 47″ N 131°06′ 51″ E	Suifenhe	Andesite	106±1	Ref[26]
31	TH7-20	Linjiang	41°58′ 19″ N 127°00′ 20″ E	Ergulazi	Pyroxene andesite	131±4	Ref[27]
32	LJ18-1	Linjiang	41°43′ 07″ N 127°04′ 27″ E	Sidaogou	Rhyolite	113±4	Ref[27]
33	JD14	Jiamusi	46°28′ 20″ N 130°22′ 45″ E	Dyke	Rhyolite	100±2	Ref[28]
34	JD07	Jiamusi	46°28′ 20″ N 130°22′ 45″ E	Dyke	Dolerite	100±2	Ref[28]
35	ML07-1	Muleng	44°45′ 29″ N 130°42′ 36″ E	Yilin	Rhyolite	104±1	Ref[28]
36	HG02-4	Hegang	47°19′ 39″ N 130°22′ 44″ E	Shihougou	Tuff	100±2	Ref[28]
37	HN14-2	Huanan	46°19′ 39″ N 130°58′ 23″ E	Dyke	granite porphyry	100±2	Ref[28]
38	HN18-6	Huanan	46°19′ 39″ N 130°58′ 23″ E	Dyke	Andesite	100±1	Ref[28]
39	14GW138	Dongshan	46°15′ 46″ N 133°09′ 28″ E	Dalingqiao	Rhyolite	182±4	Ref[29]
40	14GW184	Qindeli	48°04′ 57″ N 133°18′ 09″ E	Dalingqiao	Rhyolite	176±3	Ref[29]
41	14GW144	Haiyinshan	46°23′ 42″ N 133°13′ 54″ E	Dababeishan	Rhyolitic tuff	174±3	Ref[29]
42	14GW152	Qingshankou	46°28′ 28″ N 133°17′ 46″ E	Dajiahe	Rhyolitic tuff	187±4	Ref[29]
43	14GW177	Qindeli	48°04′ 57″ N 133°18′ 09″ E	Dalingqiao	Andesite	174±2	Ref[29]

References

1. Zhang, J. H., Ge, W. C., Wu, F. Y., Wilde, S. A., Yang, J. H. & Liu, X. M. Large-scale Early Cretaceous volcanic events in the northern Great Xing'an Range, northeastern China. *Lithos* **102**, 138–157 (2008).

2. Zhang, J. H., Gao, S., Ge, W. C., Wu, F. Y., Yan, J. H., Wilde, S. A. & Li, M. Geochronology of the Mesozoic volcanic rocks in the Great Xing'an Range, northeastern China: Implications for subduction-induced delamination. *Chem. Geol.* **276**, 144–165 (2010).

3. Ying, J. F., Zhou, X. H. & Zhang, L. C., Wang, F. Geochronological framework of Mesozoic volcanic rocks in the Great Xing'an Range, NE China, and their geodynamic implications. *J. Asian Earth Sci.* **39**, 786–793 (2010).

4. Li, P. P., Ge, W. C. & Zhang, Y. L. Division of volcanic strata in the northwestern part of

Hailar basin: Evidence from zircon U–Pb dating. *Acta Petrol. Sin.* **26**, 2482–2494 (2010).

5. Meng, E., Xu, W. L., Yang, D. B., Qiu, K. F., Li, C. H. & Zhu, H. T. Zircon U–Pb chronology, geochemistry of Mesozoic volcanic rocks from the Lingquan basin in Manzhouli area, and its tectonic implications. *Acta Petrol. Sin.* **27**, 1209–1226 (2011).
6. Xu, M. J., Xu, W. L., Meng, E. & Wang, F. Zircon U–Pb chronology andgeochemistry of Mesozoic volcanic rocks from the Shanghulin–Xiangyang basins in Erguna area, and its tectonic implications. *Geol. Bull. China* **30**, 1321–1338 (2011).
7. Sun, D. Y., Gou, J., Ren, Y. S., Fu, C. L., Wang, X. & Liu, X. M. Zircon U–Pb dating and study on geochemistry of volcanic rocks in Manitu Formation from southern Manchuria, Inner Mongolia. *Acta Petrol. Sin.* **10**, 3083–3094 (2011).
8. Zhao, Z. H., Sun, D. Y., Gou, J., Ren, Y. S., Fu, C. L., Zhang, X. Y., Wang, X. & Liu, X. M. Chronology and geochemistry of volcanic rocks in Tamulangou Formation from southern Manchuria, Inner-Mongolia. *Journal of Jilin University (Earth Science Edition)* **6**, 1865–1880 (2011).
9. Gou, J., Sun, D. Y., Zhao, Z. H., Ren, Y. S., Zhang, X. Y., Fu, C. L., Wang, X. & Wei, H. Y. Zircon LA-ICPMS U–Pb dating and petrogenesis of rhyolite in Baiyingaolao Formation from the southern Manzhouli, Inner-Mongolia. *Acta Petrol. Sin.* **26**, 333–344 (2010).
10. Xu, W. L., Pei, F. P., Wang, F., Meng, E., Ji, W. Q., Yang, D. B. & Wang, W. Spatial–temporal relationships of Mesozoic volcanic rocks in NE China: constraints on tectonic overprinting and transformations between multiple tectonic systems. *J. Asian Earth Sci.* **74**, 167–193 (2013).
11. Gou, J., Sun, D. Y., Liu, Y. J., Ren, Y. S., Zhao, Z. H. & Liu, X. M. Geochronology, petrogenesis, and tectonic setting of Mesozoic volcanic rocks, southern Manzhouli area, Inner Mongolia. *Int. Geol. Rev.* **55**, 1029–1048 (2013).
12. Dong, Y., Ge, W. C., Yang, H., Zhao, G. C., Wang, Q. H., Zhang, Y. L. & Su, L.

Geochronology and geochemistry of Early Cretaceous volcanic rocks from the Baiyingaolao Formation in the central Great Xing'an Range, NE China, and its tectonic implications. *Lithos* **205**, 168–184 (2014).

13. Yang, W. B., Niu, H. C., Cheng, L. R., Shan, Q. & Li, N. B. Geochronology, geochemistry and geodynamic implications of the Late Mesozoic volcanic rocks in the southern Great Xing'an Mountains, NE China. *J. Asian Earth Sci.* **113**, 454– 470 (2015).
14. Ji, Z., Ge, W. C., Wang, Q. H., Yang, H., Zhao, G. C., Bi, J. H. & Dong, Y. Petrogenesis of Early Cretaceous volcanic rocks of the Manketouebo Formation in the Wuchagou region, central Great Xing'an Range, NE China, and tectonic implications: geochronological, geochemical, and Hf isotopic evidence. *Int. Geol. Rev.* **58**, 556–573 (2016).
15. Gu, A. L., Sun, J. G., Bai, L. A., Zhang, Y., Cui, P. L., Chai, P., Zhao, K. Q., Sun, L., Ren, L., Chen, Y. J. & Zhu, J. Q. Petrogenesis and geodynamic significance of the Ganhe Formation lavas, eastern Great Xing'an Range, China: Evidence from geochemistry and geochronology. *Island Arc* **25**, 87–110 (2016).
16. Ji, Z., Ge, W. C., Yang, H., Wang, Q. H., Zhang, Y. L., Wang, Z. H. & Bi, J. H. Late Jurassic rhyolites from the Wuchagou region in the central Great Xing'an Range, NE China: Petrogenesis and tectonic implications. J. Asian Earth Sci. 1**58**, 381–397 (2018).
17. Zhao, L., Gao, F. H., Zhang, Y. L., Xu. H. M., & Zhang, L. Y. Zircon U–Pb chronology and its geological implications of Mesozoic volcanic rocks from the Hailaer basin. *Acta Petrol. Sin.* **29**, 864–874 (2013).
18. Pei, F. P., Xu, W. L., Yang, D. B., Ji, W. Q., Yu, Y. & Zhang, X.Z. Mesozoic volcanic rocks in the southern Songliao basin: zircon U–Pb ages and their constraints on the nature of basin basement. *Earth Science* **5**, 603–617 (2008).
19. Zhang, F. Q., Pang, Y. M., Yang, S. F., Dong, C. W., Chen, H. L. & Shu, P. Geochronologyof zircon SHRIMP, geochemistry and its implication of the volcanic rocks

from Yingcheng Formation in depression area, the northern Songliao Basin. *Acta Petrol. Sin.* **81**, 1248–1258 (2007).

20. Zhang, F. Q., Cheng, X. G., Chen, H. L., Dong, C. W., Yu, X., Xiao, J., Xu, Y., Pang, Y. M. & Shu, P. Zircon chronological and geochemical constraints on the Late Mesozoic volcanic events in the southeastern margin of the Songliao Basin, NE China. *Acta Petrol. Sin.* **25**, 1–16 (2009).
21. Zhang, F. Q., Chen, H. L., Dong, C. W., Pang, Y. M., Shu, P., Wang, Y. L. & Yang, F. Shrimp zircon U–Pb geochronology of volcanic rocks and discussion on the geological time of the Yingcheng Formation of the northern Songliao Basin. *Journal of Stratigraphy* **32**, 15–20 (2008).
22. Li, S. Q., Hegner, E., Yang, Y. Z., Wu, J. D. & Chen, F. K. Age constraints on late Mesozoic lithospheric extension and origin of bimodal volcanic rocks from the Hailar basin, NE China. *Lithos* **190**, 204–219 (2014).
23. Jin, X., Ge W. C., Xue Y. F. & Jin, Y. D. Zircon U–Pb ages and Hf isotopic composition of volcanic rocks from Well Linshen 3 in Songliao Basin. *Global Geology* **30**, 7–17 (2011).
24. Zhang, F. Q., Chen, H. L., Yu, X., Dong, C. W., Yang, S. F., Pang, Y. M. & Batt, G. E. Early Cretaceous volcanism in the northern Songliao Basin, NE China, and its geodynamic implication. *Gondwana Res.* **19**, 163–176 (2011).
25. Shu, P., Ding, R. X., Ji, X. Y. & Qu, Y. M. SHRIMP zircon geochronology of reservoir volcanic rocks in the Qingshen gas field, Songliao Basin. *Acta petrologica et Mineralogica* **26**, 239–246 (2007).
26. Ji, W. Q., Xu, W. L., Yang, D. B., Pei, F. P., Jin, K. & Liu, X. M. Chronology and geochemistry of volcanic rocks in the Cretaceous Suifenhe formation in eastern Heilongjiang, *China. Acta Petrol. Sin.* **81**, 266–277 (2007).
27. Yu, Y., Xu, W. L., Pei, F. P., Yang, D. B. & Zhao, Q. G. Chronology and geochemistry of

Mesozoic volcanic rocks in the Linjiang area, Jilin province and their tectonic implications. *Acta Petrol. Sin.* **83**, 245–257 (2009).

28. Sun, M.D. Late Mesozoic magmatism and its tectonic implication for the Jiamusi Block and adjacent areas of NE China (Ph.D dissertation). Zhejiang University (2013).

29. Wang, Z. H., Ge, W. C., Yang, H., Bi, J. H., Ji, Z., Dong, Y. & Xu, W. L. Petrogenesis and tectonic implications of Early Jurassic volcanic rocks of the Raohe accretionary complex, NE China. *J. Asian Earth Sci.* **134**, 262–280 (2017).

Table S2. LA-ICP-MS zircon U-Pb dating data for late Mesozoic volcanic rocks in the Hailar Basin, NE China.

Sample no.	Th	U	Pb	Th/U	Isotopic ratios						Ages(Ma)					
	ppm	ppm	ppm		$^{207}Pb/^{206}Pb$		$^{207}Pb/^{235}U$		$^{206}Pb/^{238}U$		$^{207}Pb/^{206}Pb$		$^{207}Pb/^{235}U$		$^{206}Pb/^{238}U$	
					Ratio	1σ	Ratio	1σ	Ratio	1σ	Ages	1σ	Ages	1σ	Ages	1σ
C4-1-1	222	199	7	1.12	0.04807	0.00779	0.17482	0.02678	0.02638	0.00149	103	232	164	23	168	9
C4-1-2	163	128	6	1.27	0.05276	0.02065	0.21161	0.08221	0.02909	0.00146	318	615	195	69	185	9
C4-1-3	178	183	7	0.97	0.04732	0.00635	0.16882	0.02141	0.02587	0.00120	65	193	158	19	165	8
C4-1-4	153	159	6	0.96	0.05191	0.00924	0.18586	0.03190	0.02596	0.00130	281	287	173	27	165	8
C4-1-5	145	178	6	0.81	0.05127	0.00811	0.17970	0.02792	0.02542	0.00083	253	283	168	24	162	5
C4-1-6	327	240	10	1.37	0.04916	0.00786	0.17225	0.02703	0.02541	0.00083	155	267	161	23	162	5
C4-1-7	206	215	8	0.96	0.06022	0.00551	0.19800	0.01699	0.02384	0.00082	611	126	183	14	152	5
C4-1-8	143	174	6	0.82	0.05465	0.00568	0.18913	0.01869	0.02510	0.00087	398	160	176	16	160	5
C4-1-9	163	191	7	0.85	0.04835	0.00600	0.19145	0.02186	0.02871	0.00148	116	160	178	19	182	9
C4-1-10	278	248	9	1.12	0.04810	0.00482	0.16697	0.01574	0.02517	0.00093	104	141	157	14	160	6
C4-1-11	96	127	5	0.76	0.05033	0.00673	0.18065	0.02289	0.02602	0.00118	210	201	169	20	166	7
C4-1-12	193	184	7	1.05	0.05168	0.01956	0.17318	0.06439	0.02430	0.00178	271	543	162	56	155	11
C4-1-13	241	203	8	1.19	0.05256	0.00441	0.18706	0.01431	0.02581	0.00096	310	107	174	12	164	6
C4-1-14	178	187	9	0.95	0.05062	0.00649	0.20259	0.02538	0.02902	0.00087	224	226	187	21	184	5
C4-1-15	112	142	5	0.79	0.05084	0.00451	0.17666	0.01441	0.02520	0.00095	234	118	165	12	160	6
C4-1-16	143	164	6	0.87	0.04605	0.00531	0.15622	0.01734	0.02460	0.00076		229	147	15	157	5
C4-1-17	90	94	3	0.96	0.05417	0.00811	0.18071	0.02539	0.02419	0.00132	378	216	169	22	154	8
C4-1-18	179	214	8	0.84	0.05099	0.00324	0.18440	0.01126	0.02623	0.00055	240	102	172	10	167	3
C4-1-19	204	207	8	0.99	0.04982	0.00355	0.17610	0.01180	0.02564	0.00069	187	104	165	10	163	4
C4-1-20	382	273	11	1.40	0.05483	0.00415	0.18753	0.01366	0.02481	0.00058	405	121	175	12	158	4
C4-2-1	195	236	10	0.83	0.06001	0.01174	0.19108	0.03659	0.02309	0.00093	604	415	178	31	147	6
C4-2-2	268	301	11	0.89	0.05547	0.01161	0.18363	0.03747	0.02401	0.00113	431	417	171	32	153	7
C4-2-3	235	287	9	0.82	0.04605	0.00405	0.15362	0.01207	0.02420	0.00095		192	145	11	154	6
C4-2-4	116	162	6	0.72	0.04875	0.00593	0.15463	0.01829	0.02301	0.00065	136	257	146	16	147	4
C4-2-5	417	306	13	1.36	0.04743	0.00851	0.15599	0.02740	0.02385	0.00086	71	318	147	24	152	5
C4-2-6	245	240	8	1.02	0.06078	0.00628	0.20324	0.01892	0.02425	0.00115	631	121	188	16	154	7
C4-2-7	140	175	6	0.80	0.05369	0.01101	0.17785	0.03552	0.02402	0.00112	358	398	166	31	153	7
C4-2-8	269	341	10	0.79	0.04893	0.00224	0.16076	0.00679	0.02383	0.00050	144	60	151	6	152	3
C4-2-9	236	245	11	0.96	0.06349	0.01568	0.18654	0.04516	0.02131	0.00104	725	518	174	39	136	7
C4-2-10	334	306	12	1.09	0.05113	0.00568	0.16614	0.01807	0.02357	0.00054	247	251	156	16	150	3
C4-2-11	176	241	7	0.73	0.04911	0.00290	0.16371	0.00891	0.02418	0.00062	153	79	154	8	154	4
C4-2-12	176	182	7	0.97	0.05424	0.01410	0.16660	0.04234	0.02228	0.00122	381	485	156	37	142	8
C4-2-13	78	97	4	0.81	0.04947	0.00860	0.16649	0.02822	0.02441	0.00095	170	326	156	25	155	6
C4-2-14	223	285	9	0.78	0.06482	0.00312	0.21427	0.00928	0.02398	0.00058	768	52	197	8	153	4
C4-2-15	240	186	7	1.29	0.06427	0.00414	0.21666	0.01259	0.02445	0.00075	751	72	199	11	156	5

C4-2-16	159	206	6	0.77	0.05356	0.00548	0.17736	0.01760	0.02402	0.00060	353	234	166	15	153	4
C4-2-17	169	202	6	0.83	0.05600	0.00995	0.17847	0.03078	0.02311	0.00099	452	377	167	27	147	6
C4-2-18	266	300	10	0.89	0.04942	0.00488	0.15582	0.01495	0.02287	0.00054	168	224	147	13	146	3
C4-2-19	213	261	8	0.81	0.06160	0.00384	0.19949	0.01123	0.02349	0.00070	660	71	185	10	150	4
WD2-01	266	163	6	1.63	0.04562	0.00315	0.15320	0.00992	0.02436	0.00064	-22	90	145	9	155	4
WD2-02	62	51	2	1.22	0.05367	0.00704	0.19501	0.02448	0.02636	0.00106	357	209	181	21	168	7
WD2-03	220	244	8	0.90	0.04944	0.00262	0.16621	0.00816	0.02439	0.00055	169	73	156	7	155	3
WD2-04	341	277	11	1.23	0.05042	0.00205	0.18418	0.00696	0.02649	0.00048	214	54	172	6	169	3
WD2-05	265	254	9	1.04	0.04921	0.00246	0.17586	0.00810	0.02592	0.00057	158	67	164	7	165	4
WD2-06	108	85	3	1.26	0.05266	0.00481	0.17651	0.01531	0.02431	0.00075	314	141	165	13	155	5
WD2-07	205	171	6	1.20	0.04442	0.00272	0.14946	0.00849	0.02440	0.00061	-49	75	141	7	155	4
WD2-08	109	72	3	1.52	0.04936	0.00494	0.16593	0.01576	0.02438	0.00081	165	152	156	14	155	5
WD2-09	64	47	2	1.36	0.04216	0.00798	0.14810	0.02668	0.02548	0.00153	-168	210	140	24	162	10
WD2-10	777	436	16	1.78	0.04422	0.00260	0.14310	0.00781	0.02347	0.00056	-59	73	136	7	150	4
WD2-11	273	184	7	1.48	0.03696	0.00290	0.12680	0.00945	0.02488	0.00067	-474	293	121	9	158	4
WD2-12	241	151	6	1.60	0.04896	0.00282	0.16468	0.00889	0.02439	0.00056	146	82	155	8	155	4
WD2-13	262	223	8	1.17	0.04262	0.00208	0.14347	0.00658	0.02441	0.00048	-143	68	136	6	155	3
WD2-14	193	101	5	1.90	0.04791	0.00569	0.17299	0.01969	0.02618	0.00094	95	186	162	17	167	6
WD2-15	337	299	10	1.13	0.04097	0.00180	0.13284	0.00544	0.02352	0.00044	-234	60	127	5	150	3
WD2-16	55	52	2	1.07	0.06237	0.00913	0.20186	0.02744	0.02347	0.00132	687	196	187	23	150	8
WD2-17	702	403	16	1.74	0.04338	0.00176	0.14535	0.00546	0.02430	0.00045	-103	51	138	5	155	3
WD2-18	339	231	9	1.47	0.04223	0.00212	0.13645	0.00642	0.02343	0.00048	-164	69	130	6	149	3
WD2-19	78	56	2	1.39	0.04050	0.00596	0.13037	0.01842	0.02335	0.00101	-261	184	124	17	149	6
GW065-01	54	52	2	1.04	0.04818	0.01252	0.14835	0.03812	0.02233	0.00086	108	439	140	34	142	5
GW065-02	208	190	7	1.09	0.04924	0.00544	0.16703	0.01803	0.02460	0.00061	159	196	157	16	157	4
GW065-03	56	48	2	1.17	0.04873	0.01071	0.16047	0.03462	0.02388	0.00109	135	324	151	30	152	7
GW065-04	81	58	2	1.39	0.04925	0.01687	0.16268	0.05471	0.02395	0.00166	160	471	153	48	153	10
GW065-05	112	85	3	1.31	0.04861	0.00701	0.16251	0.02294	0.02425	0.00076	129	246	153	20	154	5
GW065-06	126	122	5	1.03	0.04898	0.00674	0.16840	0.02271	0.02493	0.00073	147	242	158	20	159	5
GW065-07	112	138	6	0.82	0.05285	0.01162	0.17052	0.03666	0.02340	0.00116	322	350	160	32	149	7
GW065-08	87	54	2	1.61	0.06628	0.01058	0.21388	0.03319	0.02340	0.00093	815	265	197	28	149	6
GW065-09	194	217	8	0.90	0.04826	0.00440	0.16075	0.01430	0.02416	0.00050	112	159	151	13	154	3
GW065-10	124	108	4	1.15	0.04874	0.00689	0.16143	0.02236	0.02402	0.00072	135	246	152	20	153	5
GW065-11	59	50	2	1.18	0.04983	0.01121	0.16668	0.03673	0.02426	0.00119	187	333	157	32	155	7
GW065-12	56	52	2	1.09	0.04506	0.00983	0.15090	0.03225	0.02429	0.00116	-16	286	143	28	155	7
GW065-13	190	159	6	1.19	0.04927	0.00555	0.16620	0.01829	0.02447	0.00063	161	199	156	16	156	4
GW065-14	74	56	2	1.31	0.04969	0.00895	0.16555	0.02930	0.02416	0.00088	181	292	156	26	154	6
GW065-15	38	37	1	1.03	0.04906	0.01831	0.16219	0.05974	0.02398	0.00154	151	529	153	52	153	10
GW065-16	186	151	6	1.23	0.05076	0.00669	0.16194	0.02085	0.02314	0.00070	230	234	152	18	147	4
GW065-17	76	75	3	1.03	0.04754	0.00782	0.16070	0.02594	0.02452	0.00083	76	263	151	23	156	5
GW065-18	83	75	18	1.11	0.04944	0.01309	0.16092	0.04175	0.02361	0.00133	169	377	152	37	150	8
GW065-19	132	122	4	1.09	0.05010	0.00634	0.16935	0.02094	0.02452	0.00070	200	225	159	18	156	4

GW065-20	237	147	7	1.61	0.04983	0.01076	0.16791	0.03549	0.02444	0.00117	187	322	158	31	156	7
GW065-21	392	317	11	1.23	0.04886	0.00654	0.16455	0.02154	0.02443	0.00073	141	235	155	19	156	5
GW065-22	365	304	11	1.20	0.04906	0.00503	0.16101	0.01614	0.02380	0.00056	151	180	152	14	152	4
GW065-23	133	132	6	1.01	0.05414	0.01041	0.17266	0.03291	0.02313	0.00060	377	394	162	28	147	4
GW065-24	133	94	3	1.41	0.04968	0.00694	0.16208	0.02224	0.02366	0.00065	180	251	153	19	151	4
GW065-25	405	445	15	0.91	0.05142	0.00460	0.16776	0.01476	0.02366	0.00038	260	205	157	13	151	2
08GW020-01	438	1176	63	0.37	0.05470	0.00279	0.29224	0.01351	0.03871	0.00091	400	62	260	11	245	6
08GW020-02	275	292	9	0.94	0.05281	0.00727	0.15728	0.02038	0.02158	0.00104	321	203	148	18	138	7
08GW020-03	375	239	9	1.56	0.05025	0.00679	0.14521	0.01888	0.02094	0.0008	207	222	138	17	134	5
08GW020-04	1041	1345	33	0.77	0.04718	0.00533	0.133	0.01335	0.02044	0.00109	58	126	127	12	130	7
08GW020-05	616	404	14	1.53	0.05205	0.00455	0.14986	0.01196	0.02088	0.00077	288	116	142	11	133	5
08GW020-06	103	102	3	1.01	0.05508	0.0094	0.1623	0.02636	0.02137	0.00115	415	266	153	23	136	7
08GW020-07	193	183	6	1.06	0.05281	0.00597	0.15348	0.01554	0.02108	0.00109	321	138	145	14	134	7
08GW020-08	395	409	13	0.97	0.05323	0.00497	0.15598	0.01282	0.02126	0.00097	339	106	147	11	136	6
08GW020-09	127	135	4	0.94	0.05435	0.00725	0.15641	0.01932	0.02088	0.00108	386	184	148	17	133	7
08GW020-10	97	124	4	0.78	0.05231	0.00645	0.15065	0.01763	0.02089	0.00084	299	190	142	16	133	5
08GW020-11	98	150	4	0.65	0.04811	0.00645	0.13966	0.01699	0.02106	0.00122	105	166	133	15	134	8
08GW020-12	222	193	7	1.15	0.0562	0.00695	0.16484	0.01895	0.02128	0.00099	460	173	155	17	136	6
08GW020-13	138	138	4	1.00	0.04906	0.00608	0.14971	0.01773	0.02213	0.00084	151	194	142	16	141	5
08GW020-14	183	152	5	1.21	0.05	0.00979	0.14378	0.02604	0.02086	0.00158	195	262	136	23	133	10
08GW020-15	159	125	4	1.27	0.04886	0.00583	0.14496	0.01589	0.02152	0.00104	141	157	137	14	137	7
08GW020-16	199	182	7	1.09	0.0467	0.00586	0.13892	0.01527	0.02157	0.00133	34	133	132	14	138	8
08GW020-17	152	139	5	1.10	0.05124	0.00557	0.17616	0.01752	0.02493	0.00112	252	144	165	15	159	7
08GW020-18	181	318	9	0.57	0.04504	0.0049	0.13239	0.01283	0.02131	0.00108	-17	121	126	12	136	7
08GW020-19	304	528	14	0.58	0.04253	0.00307	0.12638	0.00811	0.02154	0.00074	-148	84	121	7	137	5
08GW020-20	105	99	3	1.06	0.05051	0.00916	0.17559	0.02841	0.0252	0.0021	219	215	164	25	160	13
08GW020-21	272	262	8	1.04	0.05011	0.00807	0.14745	0.02061	0.02132	0.00174	200	172	140	18	136	11
08GW020-22	751	749	23	1.00	0.04335	0.00377	0.12973	0.00991	0.02168	0.00093	-105	93	124	9	138	6
08GW043-1	186	134	4	1.39	0.06121	0.00535	0.1887	0.01504	0.02235	0.00086	647	106	176	13	142	5
08GW043-2	287	150	6	1.92	0.0734	0.00507	0.26262	0.01633	0.02595	0.00086	1025	73	237	13	165	5
08GW043-3	142	185	5	0.77	0.04884	0.0029	0.14436	0.00813	0.02144	0.00047	140	88	137	7	137	3
08GW043-4	1201	660	22	1.82	0.04796	0.00219	0.14392	0.00606	0.02176	0.00046	97	59	137	5	139	3
08GW043-5	275	236	8	1.16	0.0503	0.0038	0.1761	0.01234	0.02539	0.0008	209	104	165	11	162	5
08GW043-6	964	1007	28	0.96	0.0487	0.00097	0.14464	0.00267	0.02155	0.00028	133	21	137	2	137	2
08GW043-7	147	150	4	0.98	0.07637	0.00348	0.21612	0.00878	0.02053	0.00049	1105	45	199	7	131	3
08GW043-8	410	544	14	0.75	0.05945	0.00262	0.17098	0.00687	0.02087	0.00045	584	51	160	6	133	3
08GW043-9	223	217	6	1.03	0.06694	0.00853	0.19091	0.02358	0.02069	0.00065	836	280	177	20	132	4
08GW043-10	222	265	7	0.84	0.05278	0.00316	0.15277	0.00848	0.021	0.00053	319	81	144	7	134	3
08GW043-11	133	90	3	1.48	0.0589	0.00614	0.1703	0.01631	0.02098	0.00092	563	133	160	14	134	6
08GW043-12	79	191	12	0.41	0.05407	0.00149	0.40021	0.01016	0.05372	0.00085	374	30	342	7	337	5
08GW043-13	725	1076	45	0.67	0.05378	0.00081	0.25847	0.00362	0.03488	0.00042	362	14	233	3	221	3
08GW043-14	202	188	5	1.07	0.05214	0.00337	0.14778	0.00888	0.02057	0.00054	292	89	140	8	131	3

08GW043-15	599	343	14	1.75	0.07391	0.00215	0.26939	0.00697	0.02646	0.00046	1039	26	242	6	168	3
08GW043-16	438	282	11	1.55	0.05261	0.00691	0.1917	0.02297	0.02645	0.0015	312	168	178	20	168	9
08GW043-17	117	162	4	0.72	0.04925	0.00374	0.13933	0.0099	0.02053	0.0006	160	108	132	9	131	4
08GW043-18	100	94	3	1.06	0.06062	0.00607	0.21065	0.01954	0.02522	0.00102	626	131	194	16	161	6
08GW043-19	132	134	4	0.98	0.05059	0.00751	0.1472	0.02033	0.02112	0.00121	222	206	139	18	135	8
08GW043-20	91	155	12	0.59	0.05362	0.002	0.46646	0.01601	0.06315	0.00119	355	44	389	11	395	7
08GW047-01	254	431	10	0.59	0.04324	0.00222	0.10736	0.00516	0.01802	0.00038	-110	69	104	5	115	2
08GW047-02	274	258	7	1.06	0.06327	0.00345	0.16107	0.00793	0.01847	0.00048	717	61	152	7	118	3
08GW047-03	150	206	5	0.73	0.05585	0.00368	0.14652	0.00897	0.01904	0.00051	446	89	139	8	122	3
08GW047-04	861	583	16	1.48	0.05276	0.00233	0.13195	0.00531	0.01815	0.00038	318	54	126	5	116	2
08GW047-05	97	124	3	0.79	0.06396	0.00699	0.16368	0.01639	0.01856	0.00085	740	135	154	14	119	5
08GW047-06	63	111	3	0.57	0.06341	0.00751	0.1609	0.01785	0.0184	0.00081	722	162	151	16	118	5
08GW047-07	242	402	10	0.60	0.04648	0.00223	0.11905	0.00532	0.01858	0.00037	23	59	114	5	119	2
08GW047-08	150	294	7	0.51	0.04968	0.00226	0.12905	0.00542	0.01883	0.00038	180	61	123	5	120	2
08GW047-09	356	375	10	0.95	0.04804	0.00312	0.12272	0.00737	0.01852	0.0005	101	85	118	7	118	3
08GW047-10	116	158	4	0.73	0.06601	0.00517	0.16761	0.01218	0.01841	0.00057	807	100	157	11	118	4
08GW047-11	399	537	13	0.74	0.04749	0.00234	0.11643	0.00534	0.01777	0.00037	74	65	112	5	114	2
08GW047-12	261	289	7	0.90	0.06491	0.00381	0.16104	0.00862	0.01799	0.00047	771	69	152	8	115	3
08GW047-13	239	336	8	0.71	0.04838	0.0028	0.12585	0.00683	0.01886	0.00042	118	82	120	6	120	3
08GW047-14	282	417	10	0.68	0.05645	0.00267	0.14597	0.00638	0.01875	0.00039	470	60	138	6	120	2
08GW047-15	149	317	7	0.47	0.04958	0.00259	0.12665	0.00623	0.01854	0.00038	175	76	121	6	118	2
08GW047-16	175	222	8	0.79	0.07116	0.0077	0.22287	0.02318	0.02271	0.00068	962	231	204	19	145	4
08GW047-17	195	286	7	0.68	0.05276	0.00334	0.13005	0.0076	0.0179	0.00047	318	85	124	7	114	3
08GW047-18	128	238	6	0.54	0.04876	0.00387	0.12426	0.00925	0.01851	0.00055	136	112	119	8	118	3
08GW047-19	249	367	9	0.68	0.05851	0.00491	0.14697	0.01189	0.01822	0.0004	549	190	139	11	116	3
08GW047-20	289	388	10	0.74	0.05257	0.00309	0.13276	0.0073	0.01835	0.00043	310	83	127	7	117	3
08GW080-01	42	37	1	1.15	0.04919	0.01364	0.15075	0.04089	0.02224	0.00133	157	392	143	36	142	8
08GW080-02	221	245	6	0.90	0.05547	0.00461	0.15178	0.012	0.01986	0.00054	431	128	143	11	127	3
08GW080-03	62	55	2	1.13	0.04605	0.01203	0.1189	0.03039	0.01873	0.00102		426	114	28	120	6
08GW080-04	94	67	2	1.39	0.06894	0.0272	0.17912	0.06921	0.01885	0.00151	897	821	167	60	120	10
08GW080-05	89	116	3	0.76	0.05131	0.00576	0.13947	0.01504	0.01973	0.00065	255	182	133	13	126	4
08GW080-06	174	79	3	2.20	0.05636	0.01801	0.14741	0.04645	0.01897	0.00101	466	598	140	41	121	6
08GW080-07	334	298	8	1.12	0.05008	0.00636	0.13948	0.0173	0.0202	0.00054	199	270	133	15	129	3
08GW080-08	75	57	2	1.31	0.06473	0.01993	0.17352	0.05255	0.01944	0.00109	766	619	162	45	124	7
08GW080-09	374	191	6	1.96	0.04974	0.00453	0.13691	0.01192	0.01999	0.00057	183	143	130	11	128	4
08GW080-10	93	186	15	0.50	0.05775	0.00185	0.57872	0.01724	0.07278	0.00113	520	39	464	11	453	7
08GW080-11	139	110	3	1.26	0.05798	0.00685	0.15665	0.0174	0.01962	0.00083	529	170	148	15	125	5
08GW080-12	86	79	2	1.08	0.07642	0.01115	0.21094	0.02836	0.02005	0.00118	1106	177	194	24	128	7
08GW080-13	419	404	10	1.04	0.05152	0.00267	0.14528	0.00703	0.02048	0.00042	264	73	138	6	131	3
08GW080-14	323	356	9	0.91	0.05516	0.00274	0.15	0.00688	0.01975	0.00042	419	65	142	6	126	3
08GW080-15	131	90	2	1.46	0.04978	0.01081	0.13405	0.02794	0.01956	0.00124	185	310	128	25	125	8
08GW080-16	255	258	16	0.99	0.05406	0.00187	0.37299	0.01196	0.05012	0.00081	374	43	322	9	315	5

08GW080-17	103	117	3	0.88	0.05404	0.01106	0.14229	0.02856	0.0191	0.00076	373	400	135	25	122	5
08GW080-18	992	1098	28	0.90	0.04925	0.00159	0.15161	0.00453	0.02237	0.00033	160	43	143	4	143	2
08GW080-19	58	49	2	1.17	0.07766	0.01828	0.22103	0.04975	0.02068	0.00148	1138	351	203	41	132	9
08GW080-20	127	149	4	0.85	0.05972	0.00554	0.16212	0.01435	0.01973	0.00059	593	140	153	13	126	4
08GW080-21	357	553	17	0.64	0.05123	0.00374	0.17505	0.01243	0.02478	0.00043	251	167	164	11	158	3
08GW080-22	115	77	3	1.49	0.04605	0.00653	0.17201	0.02338	0.02709	0.00109		258	161	20	172	7
08GW080-23	342	499	12	0.69	0.04605	0.00271	0.12682	0.00703	0.01997	0.0004		129	121	6	127	3
08GW080-24	100	115	3	0.87	0.05507	0.01043	0.14506	0.02689	0.01911	0.00075	415	388	138	24	122	5
Bei39-1-1	1132	769	24	1.47	0.06101	0.00205	0.16707	0.00508	0.01986	0.00036	640	36	157	4	127	2
Bei39-1-2	185	175	5	1.05	0.06502	0.0039	0.17979	0.00973	0.02006	0.00057	775	67	168	8	128	4
Bei39-1-3	116	122	3	0.95	0.06378	0.01099	0.1729	0.02677	0.01966	0.00154	734	200	162	23	126	10
Bei39-1-4	511	911	23	0.56	0.05343	0.00259	0.14923	0.00687	0.02026	0.0003	347	112	141	6	129	2
Bei39-1-5	144	244	7	0.59	0.05861	0.00503	0.1762	0.01368	0.02181	0.00085	553	103	165	12	139	5
Bei39-1-6	473	754	19	0.63	0.06248	0.0075	0.16593	0.01915	0.01926	0.00064	691	269	156	17	123	4
Bei39-1-7	91	138	6	0.66	0.04605	0.01477	0.1413	0.0444	0.02226	0.00144		518	134	40	142	9
Bei39-1-8	147	113	5	1.30	0.05024	0.00646	0.21692	0.02562	0.03132	0.0017	206	167	199	21	199	11
Bei39-1-9	286	279	8	1.02	0.08241	0.02064	0.22088	0.05317	0.01944	0.00135	1255	554	203	44	124	9
Bei39-1-10	85	136	19	0.63	0.05612	0.0082	0.67496	0.09626	0.08723	0.00277	457	330	524	58	539	16
Bei39-1-11	140	193	13	0.73	0.06828	0.00719	0.48864	0.04612	0.05191	0.00267	877	113	404	31	326	16
Bei39-1-12	244	302	9	0.81	0.04605	0.00321	0.12859	0.00761	0.02025	0.00074		154	123	7	129	5
Bei39-1-13	155	231	7	0.67	0.05346	0.01005	0.14163	0.02594	0.01921	0.00081	348	385	134	23	123	5
Bei39-1-14	45	95	3	0.47	0.05597	0.01409	0.16119	0.03925	0.02089	0.00132	451	486	152	34	133	8
Bei39-1-15	107	123	4	0.87	0.05617	0.0172	0.14969	0.04465	0.01933	0.00133	459	578	142	39	123	8
Bei39-1-16	539	482	17	1.12	0.0543	0.01197	0.17988	0.0386	0.02403	0.00121	383	425	168	33	153	8
Bei39-1-17	532	333	11	1.60	0.06605	0.01316	0.17041	0.03322	0.01871	0.00077	808	443	160	29	120	5
Bei39-1-18	224	314	9	0.71	0.05659	0.00656	0.14981	0.01695	0.0192	0.00049	475	264	142	15	123	3
Bei39-1-19	381	843	22	0.45	0.05941	0.0028	0.17252	0.00735	0.02106	0.00049	582	53	162	6	134	3
Bei39-1-20	219	202	6	1.08	0.04605	0.00561	0.12942	0.01509	0.02038	0.00072		240	124	14	130	5

Table S3. In situ zircon Hf isotopic data for late Mesozoic volcanic rocks in the Hailar Basin, NE China.

Sample	t(Ma)	$^{176}Yb/^{177}Hf(corr)$	$^{176}Lu/^{177}Hf(corr)$	$^{176}Hf/^{177}Hf$	$2\sigma_m$	$\varepsilon_{Hf}(0)$	$\varepsilon_{Hf}(t)$	2σ	T_{DM1}（Hf）	T_{DM2}（Hf）	$f_{Lu/Hf}$
08GW020-1	136	0.015558	0.000689	0.282938	0.000022	5.88	8.8	0.8	441	627	-0.98
08GW020-2	136	0.030764	0.001334	0.282928	0.000023	5.52	8.4	0.8	463	654	-0.96
08GW020-3	136	0.144645	0.005359	0.283050	0.000032	9.82	12.3	1.1	324	401	-0.84
08GW020-4	136	0.043011	0.001799	0.282906	0.000015	4.73	7.6	0.5	502	707	-0.95
08GW020-5	136	0.013053	0.000530	0.282831	0.000029	2.10	5.0	1.0	589	869	-0.98
08GW020-6	136	0.047381	0.001833	0.282961	0.000020	6.67	9.5	0.7	423	583	-0.94
08GW020-7	136	0.029508	0.001243	0.282917	0.000029	5.13	8.0	1.0	478	679	-0.96
08GW020-8	136	0.061166	0.002358	0.283042	0.000025	9.54	12.3	0.9	309	402	-0.93
08GW020-9	136	0.041933	0.001721	0.282901	0.000022	4.56	7.4	0.8	508	718	-0.95
08GW020-10	136	0.036882	0.001527	0.282988	0.000022	7.66	10.5	0.8	379	518	-0.95
08GW020-11	136	0.025352	0.001075	0.282949	0.000020	6.25	9.1	0.7	431	606	-0.97
08GW020-12	136	0.022605	0.000923	0.282871	0.000023	3.49	6.4	0.8	540	782	-0.97
08GW020-13	136	0.035135	0.001408	0.282906	0.000020	4.73	7.6	0.7	497	705	-0.96
08GW020-14	136	0.023188	0.000997	0.282830	0.000024	2.06	5.0	0.9	598	874	-0.97
08GW020-15	136	0.044332	0.001871	0.282860	0.000021	3.12	5.9	0.7	569	811	-0.94
08GW020-16	136	0.043134	0.001816	0.282901	0.000027	4.58	7.4	1.0	509	718	-0.95
08GW020-17	136	0.029877	0.001271	0.282869	0.000019	3.42	6.3	0.7	548	788	-0.96
08GW020-18	136	0.031292	0.001370	0.282962	0.000025	6.71	9.6	0.9	416	578	-0.96
08GW020-19	136	0.027800	0.001081	0.282957	0.000026	6.55	9.4	0.9	419	587	-0.97
08GW020-20	245	0.021104	0.000878	0.282784	0.000019	0.42	5.7	0.7	662	913	-0.97
08GW043-1	135	0.029786	0.001217	0.283001	0.000025	8.12	11.0	0.9	357	488	-0.96
08GW043-2	135	0.032194	0.001640	0.282964	0.000018	6.78	9.6	0.7	416	576	-0.95
08GW043-3	166	0.023352	0.000955	0.282827	0.000023	1.93	5.5	0.8	603	864	-0.97
08GW043-4	135	0.030124	0.001274	0.282987	0.000029	7.61	10.5	1.0	378	520	-0.96
08GW043-5	135	0.071153	0.002641	0.282969	0.000024	6.97	9.7	0.9	420	570	-0.92
08GW043-6	135	0.040219	0.001720	0.282867	0.000023	3.36	6.2	0.8	557	795	-0.95
08GW043-7	135	0.036125	0.001421	0.282863	0.000020	3.23	6.1	0.7	558	802	-0.96
08GW043-8	135	0.032252	0.001342	0.283039	0.000029	9.44	12.3	1.0	304	403	-0.96
08GW043-9	135	0.046844	0.001932	0.282936	0.000020	5.81	8.6	0.7	459	639	-0.94
08GW043-10	135	0.030775	0.001270	0.282925	0.000024	5.43	8.3	0.9	467	660	-0.96
08GW043-11	135	0.037744	0.001534	0.283023	0.000028	8.87	11.7	1.0	329	441	-0.95
08GW043-12	135	0.027700	0.001152	0.282894	0.000023	4.30	7.2	0.8	511	732	-0.97
08GW043-13	135	0.037975	0.001572	0.282871	0.000019	3.49	6.3	0.7	550	787	-0.95
08GW043-14	166	0.020464	0.000903	0.282803	0.000018	1.08	4.6	0.6	636	918	-0.97
08GW043-15	135	0.017471	0.000734	0.282836	0.000025	2.27	5.2	0.9	586	860	-0.98
08GW043-16	135	0.037524	0.001529	0.282957	0.000035	6.55	9.4	1.2	424	590	-0.95
08GW043-17	135	0.048203	0.001912	0.282915	0.000035	5.06	7.9	1.2	490	688	-0.94

08GW043-18	166	0.043938	0.001727	0.282811	0.000025	1.40	4.8	0.9	637	904	-0.95
08GW047-1	118	0.045999	0.001872	0.282874	0.000020	3.59	6.0	0.7	550	791	-0.94
08GW047-2	118	0.041671	0.001762	0.282958	0.000020	6.57	9.0	0.7	426	600	-0.95
08GW047-3	118	0.027974	0.001147	0.282914	0.000024	5.01	7.5	0.8	482	696	-0.97
08GW047-4	118	0.029102	0.001227	0.282892	0.000020	4.25	6.7	0.7	514	746	-0.96
08GW047-5	118	0.076484	0.003005	0.282885	0.000024	4.01	6.4	0.8	550	770	-0.91
08GW047-6	118	0.027727	0.001158	0.282986	0.000035	7.57	10.1	1.2	379	532	-0.97
08GW047-7	118	0.033290	0.001405	0.282923	0.000023	5.35	7.8	0.8	472	676	-0.96
08GW047-8	118	0.061304	0.002523	0.283004	0.000022	8.22	10.6	0.8	366	498	-0.92
08GW047-9	118	0.093845	0.003638	0.282973	0.000022	7.11	9.4	0.8	425	574	-0.89
08GW047-10	118	0.027316	0.001171	0.282952	0.000033	6.35	8.9	1.2	428	610	-0.96
08GW047-11	118	0.025309	0.001041	0.282888	0.000031	4.09	6.6	1.1	517	755	-0.97
08GW047-12	118	0.048899	0.002013	0.282955	0.000028	6.47	8.9	1.0	433	607	-0.94
08GW047-13	118	0.049060	0.002039	0.282872	0.000023	3.52	6.0	0.8	555	796	-0.94
08GW047-14	118	0.040310	0.001641	0.282989	0.000029	7.67	10.1	1.0	379	528	-0.95
08GW047-15	118	0.034422	0.001436	0.282834	0.000019	2.19	4.7	0.7	600	879	-0.96
08GW047-16	118	0.027080	0.001123	0.282943	0.000019	6.04	8.5	0.7	440	630	-0.97
08GW047-17	118	0.033694	0.001403	0.282966	0.000024	6.85	9.3	0.8	411	580	-0.96
08GW047-18	118	0.032102	0.001311	0.282951	0.000023	6.32	8.8	0.8	431	613	-0.96
Bei39-1-1	128	0.042788	0.001815	0.282895	0.000022	4.34	7.0	0.8	518	737	-0.95
Bei39-1-2	128	0.039578	0.001645	0.282879	0.000019	3.80	6.5	0.7	538	771	-0.95
Bei39-1-3	128	0.013989	0.000610	0.282883	0.000021	3.91	6.7	0.7	519	758	-0.98
Bei39-1-4	128	0.049286	0.002202	0.282870	0.000022	3.47	6.1	0.8	560	795	-0.93
Bei39-1-5	128	0.031542	0.001360	0.282805	0.000020	1.18	3.9	0.7	640	937	-0.96
Bei39-1-6	128	0.045835	0.001843	0.282836	0.000018	2.27	4.9	0.7	603	870	-0.94
Bei39-1-7	128	0.050396	0.002238	0.282906	0.000023	4.73	7.3	0.8	508	715	-0.93
Bei39-1-8	128	0.061734	0.002606	0.282872	0.000014	3.53	6.1	0.5	563	793	-0.92
Bei39-1-9	128	0.179334	0.006963	0.282887	0.000038	4.07	6.3	1.3	615	782	-0.79
Bei39-1-10	128	0.051412	0.002096	0.282911	0.000015	4.92	7.6	0.5	498	701	-0.94
Bei39-1-11	128	0.044906	0.001851	0.282902	0.000019	4.59	7.2	0.7	508	721	-0.94
Bei39-1-12	539	0.016543	0.000699	0.282674	0.000019	-3.48	8.1	0.7	814	981	-0.98
Bei39-1-13	326	0.024950	0.001095	0.282772	0.000020	0.00	6.9	0.7	683	894	-0.97
Bei39-1-14	199	0.022698	0.001004	0.282777	0.000024	0.19	4.4	0.9	673	956	-0.97
Bei39-1-15	128	0.041751	0.001728	0.282787	0.000017	0.51	3.2	0.6	673	981	-0.95
Bei39-1-16	128	0.047990	0.002085	0.282847	0.000019	2.64	5.3	0.7	592	847	-0.94
Bei39-1-17	128	0.014110	0.000626	0.282879	0.000019	3.77	6.5	0.7	524	767	-0.98
Bei39-1-18	128	0.048840	0.002014	0.282811	0.000016	1.37	4.0	0.6	643	928	-0.94
Bei39-1-19	128	0.031131	0.001310	0.282817	0.000025	1.60	4.3	0.9	622	910	-0.96
C4-1-1	161	0.030252	0.001303	0.282882	0.000022	3.90	7.4	0.8	529	740	-0.96
C4-1-2	161	0.037383	0.001359	0.282865	0.000021	3.29	6.8	0.7	554	779	-0.96
C4-1-3	161	0.030127	0.001217	0.282818	0.000020	1.64	5.2	0.7	619	884	-0.96
C4-1-4	161	0.016074	0.000653	0.282804	0.000020	1.14	4.7	0.7	630	912	-0.98

C4-1-5	161	0.035847	0.001498	0.282898	0.000020	4.47	8.0	0.7	509	705	-0.95
C4-1-6	161	0.031842	0.001234	0.282864	0.000022	3.25	6.8	0.8	554	781	-0.96
C4-1-7	161	0.039540	0.001441	0.282869	0.000019	3.42	6.9	0.7	550	772	-0.96
C4-1-8	161	0.019531	0.000773	0.282814	0.000018	1.47	5.1	0.7	618	892	-0.98
C4-1-9	161	0.019547	0.000809	0.282833	0.000020	2.17	5.7	0.7	591	847	-0.98
C4-1-10	161	0.054189	0.002168	0.282844	0.000017	2.55	6.0	0.6	598	833	-0.93
C4-1-11	161	0.028092	0.001155	0.282855	0.000021	2.94	6.5	0.7	565	800	-0.97
C4-1-12	161	0.068726	0.002432	0.282913	0.000021	5.00	8.4	0.7	500	677	-0.93
C4-1-13	184	0.030831	0.001221	0.282834	0.000023	2.20	5.7	0.8	596	848	-0.96
C4-1-14	161	0.029654	0.001170	0.282816	0.000021	1.56	5.1	0.7	621	888	-0.96
C4-1-15	161	0.045575	0.001709	0.282836	0.000021	2.27	5.7	0.7	602	847	-0.95
C4-1-16	161	0.044806	0.001637	0.282948	0.000045	6.24	9.7	1.6	438	592	-0.95
C4-1-17	161	0.018483	0.000745	0.282803	0.000022	1.08	4.7	0.8	633	916	-0.98
C4-1-18	161	0.022218	0.000909	0.282813	0.000019	1.46	5.0	0.7	621	893	-0.97
C4-2-1	150	0.018915	0.000780	0.282831	0.000020	2.10	5.3	0.7	593	862	-0.98
C4-2-2	150	0.032491	0.001278	0.282881	0.000030	3.86	7.0	1.1	530	752	-0.96
C4-2-3	150	0.038743	0.001561	0.282882	0.000024	3.89	7.0	0.8	533	752	-0.95
C4-2-4	150	0.025260	0.000999	0.282806	0.000022	1.22	4.4	0.8	632	919	-0.97
C4-2-5	150	0.017551	0.000719	0.282807	0.000018	1.23	4.4	0.6	627	917	-0.98
C4-2-6	150	0.024021	0.000960	0.282780	0.000020	0.29	3.5	0.7	669	979	-0.97
C4-2-7	150	0.017884	0.000746	0.282810	0.000019	1.35	4.6	0.7	623	909	-0.98
C4-2-8	150	0.020071	0.000847	0.282817	0.000018	1.60	4.8	0.6	615	894	-0.97
C4-2-9	150	0.017966	0.000719	0.282846	0.000021	2.63	5.8	0.7	571	827	-0.98
C4-2-10	150	0.016164	0.000670	0.282807	0.000024	1.24	4.5	0.8	626	916	-0.98
C4-2-11	150	0.031922	0.001305	0.282832	0.000022	2.13	5.3	0.8	601	863	-0.96
C4-2-12	150	0.029164	0.001188	0.282791	0.000020	0.65	3.8	0.7	658	957	-0.96
C4-2-13	150	0.045992	0.001757	0.282724	0.000019	-1.69	1.4	0.7	764	1110	-0.95
C4-2-14	150	0.015392	0.000651	0.282818	0.000019	1.63	4.9	0.7	610	891	-0.98
C4-2-15	150	0.023976	0.000941	0.282793	0.000032	0.74	3.9	1.1	650	950	-0.97
C4-2-16	150	0.019126	0.000811	0.282816	0.000025	1.56	4.8	0.9	615	896	-0.98
C4-2-17	150	0.028353	0.001130	0.282790	0.000021	0.63	3.8	0.8	658	958	-0.97
C4-2-18	150	0.023945	0.000974	0.282764	0.000025	-0.29	2.9	0.9	692	1016	-0.97
C4-2-19	150	0.020323	0.000834	0.282846	0.000024	2.60	5.8	0.8	574	830	-0.97
WD2-1	156	0.037397	0.001520	0.282890	0.000021	4.16	7.4	0.7	521	731	-0.95
WD2-2	156	0.038978	0.001543	0.282988	0.000026	7.64	10.9	0.9	380	508	-0.95
WD2-3	156	0.026798	0.001026	0.282861	0.000027	3.16	6.5	1.0	555	792	-0.97
WD2-4	156	0.021761	0.000943	0.282871	0.000018	3.49	6.8	0.6	540	770	-0.97
WD2-5	156	0.028197	0.001196	0.282895	0.000022	4.35	7.7	0.8	509	717	-0.96
WD2-6	156	0.038380	0.001528	0.282850	0.000019	2.75	6.0	0.7	579	821	-0.95
WD2-7	156	0.038102	0.001591	0.282891	0.000025	4.21	7.5	0.9	520	728	-0.95
WD2-8	156	0.028819	0.001193	0.282841	0.000020	2.45	5.8	0.7	586	838	-0.96
WD2-9	156	0.046824	0.001534	0.282949	0.000034	6.26	9.5	1.2	436	596	-0.95

WD2-10	156	0.031258	0.001256	0.282786	0.000029	0.51	3.8	1.0	665	963	-0.96
WD2-11	156	0.038062	0.001484	0.282862	0.000021	3.19	6.5	0.8	560	793	-0.96
WD2-12	156	0.028112	0.001155	0.282869	0.000023	3.43	6.7	0.8	546	775	-0.97
WD2-13	156	0.034731	0.001503	0.282807	0.000021	1.23	4.5	0.8	640	919	-0.95
WD2-14	156	0.039772	0.001566	0.282920	0.000020	5.24	8.5	0.7	478	662	-0.95
WD2-15	156	0.034811	0.001371	0.282910	0.000024	4.88	8.2	0.9	490	684	-0.96
WD2-16	156	0.038582	0.001585	0.282860	0.000017	3.10	6.4	0.6	565	799	-0.95
WD2-17	156	0.033733	0.001485	0.282872	0.000020	3.53	6.8	0.7	547	771	-0.96
WD2-18	156	0.035129	0.001383	0.282864	0.000022	3.24	6.5	0.8	557	789	-0.96
WD2-19	156	0.020067	0.000814	0.282819	0.000026	1.65	5.0	0.9	612	887	-0.98

Table S4. Major (wt.%) and trace (ppm) elements compositions for late Mesozoic volcanic rocks in the Hailar Basin, NE China.

Sample	08GW020	08GW043-1	08GW043-2	C4-1-1	C4-1-2	08GW047-1	08GW047-2	08GW080-1	08GW080-2	bei39-1-1
Well name	He9	Bei16	Bei16	Chu4	Chu4	Bei38	Bei38	Bei20	Bei20	bei39
Rock type	Rhyolite	Rhyolite	Rhyolite	Dacite	Dacite	Rhyolite	Rhyolite	Rhyolitic tuff	Rhyolitic tuff	Dacite
Major element (*wt.*%)										
SiO_2	80.35	70.73	73.61	72.37	72.57	72.53	73.78	71.35	70.73	69.57
TiO_2	0.17	0.45	0.37	0.16	0.16	0.23	0.23	0.34	0.35	0.57
Al_2O_3	8.38	10.17	8.53	12.87	12.49	14.09	12.53	13.67	14.37	15.06
TFe_2O_3	0.50	2.24	1.87	1.55	1.54	0.77	0.72	1.25	1.30	1.25
MnO	0.01	0.02	0.02	0.05	0.05	0.01	0.01	0.01	0.01	0.03
MgO	0.47	2.12	1.79	0.43	0.42	0.80	0.76	0.75	0.74	0.45
CaO	0.15	0.78	0.64	0.91	0.90	0.21	0.19	0.20	0.20	0.78
Na_2O	0.41	2.55	2.11	3.14	3.13	0.76	0.75	1.18	1.17	3.50
K_2O	3.47	3.25	2.68	5.52	5.63	3.13	3.10	4.36	4.47	4.46
P_2O_5	0.01	0.03	0.02	0.04	0.03	0.02	0.01	0.03	0.03	0.03
LOI	2.73	5.70	5.70	1.53	1.53	6.03	6.03	5.51	5.50	3.50
Total	96.64	98.03	97.35	98.56	98.46	98.57	98.10	98.66	98.86	99.21
Trace element (*ppm*)										
Sc	3.27	5.32	5.45	3.88	3.76	3.90	3.89	4.90	4.93	8.54
V	21.24	113.92	115.48	17.87	18.56	4.50	4.74	35.38	36.26	29.78
Cr	18.11	26.38	27.68	0.74	1.30	1.25	0.74	16.17	17.71	9.41
Co	1.77	6.42	6.65	1.14	1.16	0.91	0.89	2.65	2.65	1.86
Ni	4.74	15.36	15.37	0.65	0.97	1.35	0.80	7.57	7.19	2.15
Cu	3.29	12.60	13.30	1.48	1.29	7.15	7.66	5.56	5.16	5.24
Zn	42.60	73.74	95.30	64.66	57.92	68.18	60.34	96.64	91.66	84.52
Ga	16.75	16.63	17.27	15.41	15.75	18.57	18.12	22.94	22.94	19.28
Rb	124.2	69.1	70.6	200.8	208.9	68.5	68.8	151.9	146.8	146.9
Sr	55.0	146.5	149.9	98.1	100.5	69.8	71.0	66.4	65.1	289.0
Y	20.20	11.76	12.01	12.90	13.15	27.57	27.51	20.03	20.49	25.37
Zr	148	139	141	121	127	364	344	326	322	426
Nb	18.13	13.73	13.99	13.28	13.83	21.92	22.04	23.50	23.02	20.10
Cs	22.38	10.48	10.59	2.39	2.50	7.26	7.24	5.08	4.93	17.54
Ba	602	458	468	305	313	381	383	632	627	1052
La	17.75	21.10	21.34	43.34	45.19	47.45	47.35	46.98	45.50	46.88
Ce	44.85	49.80	50.71	80.05	83.12	101.06	104.28	92.69	89.91	97.24
Pr	5.49	4.80	4.90	7.91	8.24	10.97	10.92	9.77	9.53	10.03
Nd	19.20	15.37	15.56	22.96	23.90	36.67	36.43	29.69	28.96	31.93
Sm	3.90	2.30	2.32	3.38	3.50	6.56	6.49	4.73	4.58	5.41
Eu	0.31	0.44	0.45	0.49	0.50	0.60	0.60	0.65	0.65	1.19

Gd	3.28	1.94	1.97	2.63	2.76	5.59	5.56	3.83	3.74	4.70
Tb	0.49	0.27	0.28	0.34	0.34	0.79	0.79	0.54	0.54	0.68
Dy	3.23	1.78	1.79	1.98	2.03	4.85	4.80	3.39	3.34	4.26
Ho	0.66	0.38	0.38	0.39	0.40	0.95	0.93	0.66	0.67	0.84
Er	2.02	1.25	1.27	1.21	1.26	2.78	2.76	2.01	2.00	2.50
Tm	0.30	0.20	0.20	0.20	0.20	0.42	0.41	0.30	0.30	0.38
Yb	1.93	1.34	1.36	1.31	1.37	2.73	2.68	1.88	1.95	2.38
Lu	0.27	0.21	0.21	0.20	0.21	0.41	0.40	0.28	0.28	0.36
Hf	3.57	3.29	3.31	2.84	2.98	8.50	8.24	7.96	7.72	9.95
Ta	1.10	0.84	0.76	0.77	0.83	1.30	1.28	1.49	1.39	1.34
Pb	1.19	5.02	5.04	6.47	6.54	9.01	8.87	4.79	5.14	3.60
Th	6.12	6.77	6.71	13.40	13.82	14.25	13.83	10.49	10.07	10.83
U	2.54	1.62	1.62	2.50	2.59	2.54	2.49	3.03	2.90	3.06
Eu/Eu^*	0.26	0.62	0.62	0.48	0.48	0.30	0.30	0.45	0.47	0.70
$(La/Yb)_N$	6.19	10.63	10.60	22.25	22.21	11.73	11.90	16.82	15.70	13.26
T_{Zr}(°C)	835	784	792	760	760	947	937	912	913	897

Sample	08GW020	C4-2-1	C4-2-2	C4-2-3	WD2-1	WD2-2	GW065	AGV-2	GSR-1
Well name	He9	Chu4	Chu4	Chu4	WuD2	WuD2	WuD2		
Rock type	Rhyolite	Rhyolite	Rhyolite	Rhyolite	Rhyolite	Rhyolite	Rhyolite		
Major element (*wt.*%)									
SiO_2	80.35	75.14	75.53	75.41	71.99	71.49	70.90	60.18	72.60
TiO_2	0.17	0.18	0.18	0.18	0.37	0.40	0.44	1.05	0.29
Al_2O_3	8.38	11.14	11.05	11.28	14.58	15.00	16.10	16.43	13.73
TFe_2O_3	0.50	0.70	0.70	0.73	0.22	0.25	0.66	6.64	2.15
MnO	0.01	0.04	0.04	0.04	0.01	0.01	0.01	0.09	0.05
MgO	0.47	0.32	0.30	0.31	0.17	0.18	0.18	1.86	0.41
CaO	0.15	0.29	0.33	0.29	0.07	0.05	0.12	5.01	1.52
Na_2O	0.41	2.45	2.39	2.35	0.26	0.26	0.49	4.24	3.10
K_2O	3.47	5.46	5.14	5.14	7.37	7.59	8.22	2.96	5.02
P_2O_5	0.01	0.03	0.03	0.04	0.06	0.06	0.06	0.55	0.09
LOI	2.73	2.09	2.09	2.08	3.73	3.71	2.94		
Total	96.64	97.84	97.78	97.84	98.83	98.98	99.87		
Trace element (*ppm*)									
Sc	9.58	2.17	2.24	2.03	6.07	5.70	3.51	12.29	6.11
V	33.74	4.53	4.62	4.50	5.19	5.60	3.04	124.38	24.05
Cr	10.72	0.59	0.74	0.88	4.48	3.78	0.52	18.07	3.65
Co	2.02	0.18	0.18	0.23	0.26	0.25	0.10	16.58	3.50
Ni	2.92	0.51	0.45	0.51	2.11	1.68	0.08	19.06	2.31
Cu	5.66	1.21	1.22	1.34	1.49	1.34	1.76	53.76	3.20
Zn	96.00	151.48	151.88	169.18	75.38	60.79	36.78	85.29	27.83

Ga	20.12	19.46	19.88	18.97	32.70	29.94	21.29	21.05	19.39
Rb	158.6	141.6	138.3	133.0	289.1	259.5	220.3	68.8	444.1
Sr	299.7	193.9	198.0	190.8	136.4	125.9	110.9	653.7	97.8
Y	26.52	23.52	24.34	23.35	49.58	46.02	43.46	19.93	60.03
Zr	448	286	304	287	740	680	505	235	167
Nb	20.78	33.66	34.36	33.36	36.46	33.29	24.71	15.40	41.27
Cs	17.93	2.41	2.46	2.36	7.99	7.39	9.17	1.17	38.80
Ba	1075	204	208	203	1265	1182	1119	1140	366
La	48.13	41.63	42.17	40.79	110.03	101.24	70.29	39.69	50.94
Ce	98.15	101.27	100.98	98.49	225.48	208.58	139.90	65.53	102.28
Pr	10.24	10.92	11.07	10.70	25.00	23.26	17.60	8.08	11.46
Nd	32.32	34.29	34.55	33.25	84.45	78.49	63.71	29.96	48.61
Sm	5.47	6.49	6.55	6.29	13.30	12.37	11.03	5.98	9.35
Eu	1.21	0.28	0.28	0.27	2.73	2.53	2.46	1.54	0.87
Gd	4.69	5.15	5.24	5.01	10.36	9.64	10.19	4.65	9.34
Tb	0.69	0.74	0.75	0.71	1.45	1.36	1.61	0.66	1.66
Dy	4.26	4.44	4.48	4.27	8.66	8.07	8.77	3.61	10.23
Ho	0.84	0.84	0.85	0.81	1.63	1.52	1.70	0.70	2.06
Er	2.50	2.45	2.46	2.35	4.58	4.27	4.81	1.80	6.54
Tm	0.38	0.36	0.36	0.35	0.65	0.60	0.66	0.26	1.03
Yb	2.38	2.19	2.24	2.11	3.96	3.74	4.25	1.65	7.61
Lu	0.36	0.30	0.31	0.30	0.57	0.54	0.68	0.25	1.04
Hf	9.95	6.81	7.29	6.94	15.93	14.77	13.05	5.08	6.30
Ta	1.25	2.13	2.14	1.97	2.20	2.00	1.72	0.88	7.26
Pb	3.37	13.87	13.90	13.07	7.75	7.24	15.91	12.97	29.81
Th	10.52	7.64	7.53	7.15	6.91	6.41	19.17	6.05	54.97
U	2.96	1.95	1.92	1.81	3.64	3.37	8.15	1.86	18.35
Eu/Eu^*	0.71	0.14	0.14	0.14	0.69	0.69	0.70		
$(La/Yb)_N$	13.63	12.79	12.71	13.01	18.74	18.26	11.15		
T_{Zr}(°C)	904	849	859	857	998	987	944		
